# Overcoming Multidrug Resistance: Flavonoid and Terpenoid Nitrogen-Containing Derivatives as ABC Transporter Modulators

**DOI:** 10.3390/molecules25153364

**Published:** 2020-07-24

**Authors:** Bruno M. F. Gonçalves, David S. P. Cardoso, Maria-José U. Ferreira

**Affiliations:** Research Institute for Medicines (iMed.ULisboa), Faculty of Pharmacy, Universidade de Lisboa, 1649-003 Lisboa, Portugal; bgoncalves@farm-id.pt (B.M.F.G.); davidpcardoso@ff.ulisboa.pt (D.S.P.C.)

**Keywords:** cancer, multidrug resistance, natural products, ABC-transporters, nitrogen-containing flavonoid derivatives, nitrogen-containing terpenoid derivatives, P-gp, BCRP and MRP1

## Abstract

Multidrug resistance (MDR) in cancer is one of the main limitations for chemotherapy success. Numerous mechanisms are behind the MDR phenomenon wherein the overexpression of the ATP-binding cassette (ABC) transporter proteins P-glycoprotein (P-gp), breast cancer resistance protein (BCRP) and multidrug resistance protein 1 (MRP1) is highlighted as a prime factor. Natural product-derived compounds are being addressed as promising ABC transporter modulators to tackle MDR. Flavonoids and terpenoids have been extensively explored in this field as mono or dual modulators of these efflux pumps. Nitrogen-bearing moieties on these scaffolds were proved to influence the modulation of ABC transporters efflux function. This review highlights the potential of semisynthetic nitrogen-containing flavonoid and terpenoid derivatives as candidates for the design of effective MDR reversers. A brief introduction concerning the major role of efflux pumps in multidrug resistance, the potential of natural product-derived compounds in MDR reversal, namely natural flavonoid and terpenoids, and the effect of the introduction of nitrogen-containing groups are provided. The main modifications that have been performed during last few years to generate flavonoid and terpenoid derivatives, bearing nitrogen moieties, such as aliphatic, aromatic and heterocycle amine, amide, and related functional groups, as well as their P-gp, MRP1 and BCRP inhibitory activities are reviewed and discussed.

## 1. Introduction

Despite the fact recent advances in cancer treatment, such as targeted therapy and immunotherapy, have contributed significantly to improving the disease-free survival rate and quality of life of cancer patients, cancer is still the second cause of death and morbidity in Europe, with more than 1.9 million deaths each year [1]. The development of multidrug resistance (MDR), a phenomenon that occurs when tumors become tolerant to multiple and structurally unrelated anticancer drugs, represents one of the major obstacles to the long-term success of chemotherapy regimens. This phenomenon, that can be intrinsic, when exists before treatment, or acquired, when develops after therapy, is responsible for most cases of cancer relapses and recurrence associated with high rates of cancer-related deaths [2]. Thus, MDR is a public health problem that requests an urgent solution. However, overcoming MDR in cancer is one of the foremost challenges in the present therapeutic era since MDR is a multifactorial phenomenon involving many different mechanisms, such as over-expression of ATP-binding cassette ABC protein transporters that increased the efflux of chemotherapeutic drugs, enhanced DNA damage repair, mutation of oncogenes that become resistant to former treatments, adaptation of cancer cells to the tumor microenvironment, surviving of cancer stem cells that escape from conventional therapies, among others [3,4,5,6].

### 1.1. The Role of ABC Transporter Proteins in Cancer Multidrug Resistance

Over the past few decades, the mechanism most frequently associated with the development of MDR in cancer is the over-expression of ABC transporter proteins either at the plasma membrane or in intracellular vesicles. ABC transporters are widespread in all forms of life, with humans having 49 ABC protein-encoding genes, classified into seven subfamilies (from ABCA to ABCG based on phylogenetic similarity) that differ in size architecture and domain arrangement [7]. Among all the 49 human ABC transporters, P-glycoprotein (P-gp/ABCB1), multidrug resistance-associated protein 1 (MRP1/ABCC1) and breast cancer resistance protein (BCRP/ABCG2) are considered major players in the development of the MDR phenomenon [8,9]. These transporters, powered by ATP, act as extrusion pumps for multiple structurally and mechanistically unrelated molecules, promoting their translocation across cell membrane against their concentration gradient. ABC transporters are expressed in the plasma membrane of cells in key organs such as the intestines, liver, kidneys, blood -brain barrier and blood-placental barrier, thus affecting pharmacokinetics, toxicity and efficacy of drugs [10,11,12]. Due to their ability to transport organic and inorganic molecules across cellular membranes, including nutrients and a wide array of xenobiotics and undesirable metabolites, these transporters are essential regulators of organism homeostasis [11]. However, when overexpressed in cancer cells, ABC transporters are responsible for decreasing the intracellular accumulation of cytotoxic drugs, which results in the failure of chemotherapeutic regimens [13].

**Figure 1:**
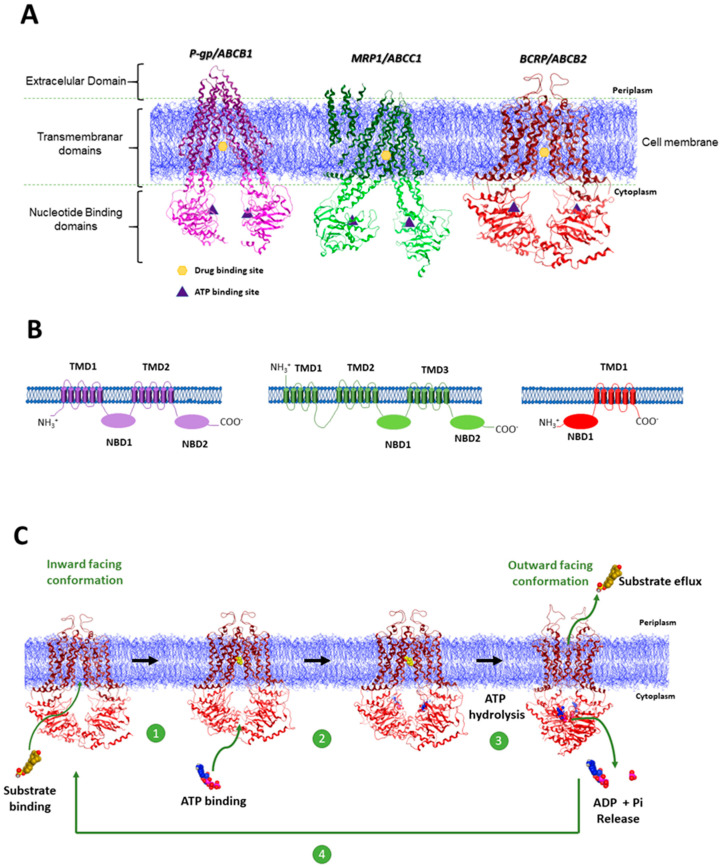
(**A**) High resolution 3D structures of the mouse P-gp (PDB-ID:5KPI) [20], which has 87% sequence identity to human P-gp; bovine MRP1(PDB-ID:5UJ9) [21], which has 91% protein identity with the human protein; and human BCRP (PDB-ID:5NJ3) [22]. The NBD domains for each protein are depicted in light purple (P-gp), light green (MRP1) and light red (BCRP), whereas the TMD domains are depicted in the dark variations of the same colors. Cell membrane is depicted in blue. Adapted from: Nat Rev Cancer 18, 452–464 (2018) [9]; (**B**) Membrane topology models of P-gp, MRP1, and BCRP proteins. P-gp possesses two homologous halves, each containing a TMD consisting of six putative membrane-spanning α-helices at the *N*-terminus and a cytosolic hydrophilic NBD at the c-terminus that are involved in binding and hydrolyzing ATP. MRP1, in addition to the two NBD and two TMD containing six α-helices, is composed by and extra TMD consisting of five α-helices. The half transporter BCRP contains one TMD of six α-helices and one NBD. The ATP-binding site of this transporter is found on the amino-terminal side (N) in contrast to P-gp and MRP1. Was adopted the same color scheme used in A. (**C**) Simplified representation of the ABC drug efflux mechanism using the 3D structure of BCRP: 1—Substrate (molecule in yellow) enters in the drug binding site localized within the TMD domains of the transporter and triggers ATP (molecules in blue and pink) binding; 2—ATP binds to the NBD of the transporter; 3—Following ATP binding is induced the formation of the NBD sandwich dimer. ATP hydrolysis triggers conformational changes resulting in an outward open TMD conformation with reduced affinity to the substrate allowing the drug extrusion. 4—ADP + Pi release induces the resetting of the transporter to its basal inward facing conformation. To build this picture were used Cryo-EM structures of BCRP: structure of BCRP with the substrate (estrone-3-sulfate) placed in the DBS (PDB-ID:6HCO) [23]; 2 ATP molecules were placed in the NBD using MOE.; structure of BCRP in the outward facing configuration and two ATP molecules placed in the NBD (PDB-ID:6HBU) [23].

Functional ABC transporters share a characteristic structural architecture that consists in a minimum of four domains: two transmembrane domains (TMD) and two nucleotide binding domains (NBD) (Figure 1A,B). TMD are constituted predominantly by hydrophobic amino acids embedded in membrane bilayer and define the translocation pathway. At a sequence level, TMD are structurally diverse which reflects the chemical diversity of the translocated substrates. The hydrophilic NBD are located at the cytoplasmic surface of the membrane and are involved in binding and hydrolyzing ATP molecules. By contrast with TMD, NBD are characterized by high conserved regions core sequence motifs, namely the A-loop (ATP binding-base stacking), Walker A and Walker B (ATP binding and hydrolysis), and a Signature C (LSGGQ) motif (phosphate binding, NBD-NBD communication) [14,15,16]. In mammals, these four domains may be encoded by a single polypeptide chain (full-transporters) or by two separate proteins (half-transporters), with the latter ones assembled as homodimers or heterodimers to create a functional transporter [15].

P-gp was the first ABC transporter to be discovered in 1976 by Juliano and Ling in Chinese hamster ovary cell mutants and is by far the most studied efflux pump. This transporter is a single polypeptide with 170 kDa characterized by two pseudo symmetric halves, each consisting of six transmembrane α-helices, embedded within the lipid bilayer, connected to one hydrophilic NBD (Figure 1B). P-gp is constitutively expressed in cells with specific barrier functions, such as intestine, liver bile ductules and kidney proximal tubules [17]. One of the characteristic features of P-gp is its broad substrate specificity ranging from small molecules, such as organic cations, carbohydrates, amino acids, and some antibiotics, to macromolecules such as polysaccharides and proteins [18]. Usually P-gp substrates are weakly amphipathic or relatively hydrophobic, and the majority contain aromatic rings and a positively charged tertiary nitrogen atom in their structure [17,19].

The MRP1 transporter was first identified in 1992 in doxorubicin H69AR lung carcinoma resistant cell line, where no P-gp expression was detected [24]. This transporter was found to be overexpressed mostly in lung cancer but also in a variety of other tumors including breast, prostate, pancreas and colon. Despite some overlapping substrate specificity between P-gp and MRP1, while P-gp recognizes mainly hydrophobic substrates, the MRP1 substrates are typically organic anions, and many of them require conjugation with glutathione (GSH), glucuronic acid, or sulfate [21]. Whereas P-gp has the typical ABC protein structure with two TMD and two functional NBD, MRP1 processes only a single functional ATP hydrolysis site and an extra TMD towards the *N*-terminus comprising five extra-transmembrane regions (TMD0) (Figure 1B) [21].

BCRP protein was discovered in 1998 in MCF-7/AdrVp cell lines [25] and in placental tissues [26]. The structural and functional understanding of this transporter remains largely behind to P-gp and MRP1. Interestingly, BCRP differs from P-gp and MRP1, since this protein is a half transporter homodimer and consists of only one cytoplasmatic NBD and one TMD with six membrane-spanning α-helices. Contrary to P-gp, which exhibits a TMD-NBD organization in both halves, BCRP has a NBD-TMD rearrangement where the NDB precedes the TMD [27]. This protein was found to be overexpressed in solid tumors as well as in hematological tumors such T/NK-cell lymphoma and diffuse large B-cell lymphoma. Like P-gp and MRP1, BCRP displays broad substrate specificity [28].

The crystallographic structures of P-gp [20,29], MRP1 [21] and BCRP [22,30], published in recent years (Figure 1A), expanded the knowledge on the proteins function at molecular level, however a comprehensive understanding of the mechanistic principles of ABC proteins efflux function remains elusive. Due to structural differences between the three efflux transporters, it is expected some level of mechanistic diversity, however the main steps of the efflux mechanism are common to all three pumps. In brief: the drug binding site (DBS) in its basal conformation (inward facing conformation) (Figure 1C) is intrinsically flexible allowing the binding of substrate molecules with high affinity and simultaneously favors the ATP binding at NBD. ATP binding triggers the NBD dimerization and the consequent ATP hydrolysis promotes a conformational change that results in the orientation of the TMD to the outside of the cell membrane (outward facing conformation), reducing the affinity of the DBS (low-affinity state) to substrate, resulting in the efflux of the substrate to the outside. The release of adenosine diphosphate (ADP) and inorganic phosphate (Pi) occurs in the final step of the efflux cycle and reconverts the transporter to its starting inward-facing conformation (Figure 1C) [15].

### 1.2. Inhibition of ABC Transporter Proteins as a Strategy to Overcome MDR

**Figure 2:**
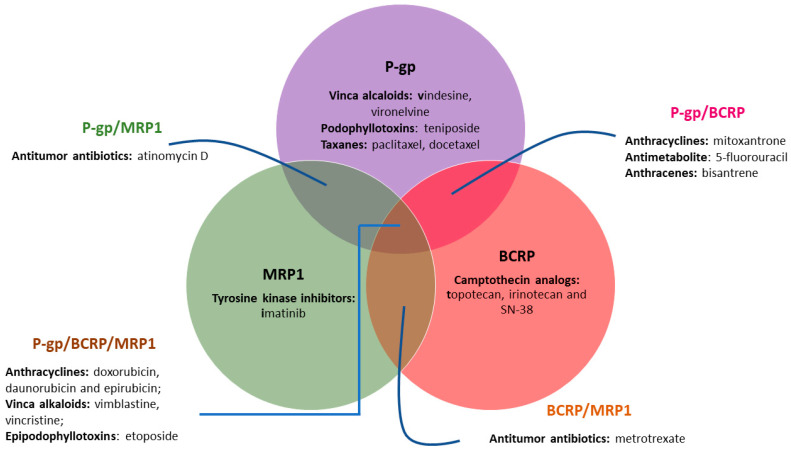
Anticancer drug substrates of P-gp, MRP1 and BCRP. As depicted in the figure, P-gp, MRP1, and BCRP have an overlapped specificity for a variety of substrates.

P-gp, MRP1, and BCRP export a broad range of structurally and mechanistically unrelated cancer chemotherapeutics, covering almost the whole range of the antineoplastic approved for clinical use, including: vinca alkaloids, podophyllotoxins, taxanes, tyrosine kinase inhibitors, camptothecin analogs, antitumor antibiotics, anthracyclines, antimetabolite, anthracenes and epipodophyllotoxins (Figure 2) [31]. Therefore, the simultaneous administration of an efficient ABC-efflux modulator/inhibitor with chemotherapeutic agents has been proposed as a powerful therapeutic strategy to overcome MDR by re-establishing the drug sensitivity of resistant cancer cells or improving the pharmacokinetics of anti-cancer agents in order to achieve better therapeutic outcomes in cancer patients. With this strategy in mind, considerable effort has been made, since the discovery of P-gp in 1976, to develop effective inhibitors towards ABC efflux transporters, involving high-throughput screenings (HTS) of commercially available drugs, together with natural products and design and synthesis of new modulators/inhibitors, which could be classified in competitive and non-competitive inhibitors. Competitive inhibitors bind tightly to the substrate binding site of the protein and block the substrate access. Differently, non-competitive inhibitors bind to a different place of the efflux pump, inhibiting or modulating its function allosterically [32].

**Figure 3:**
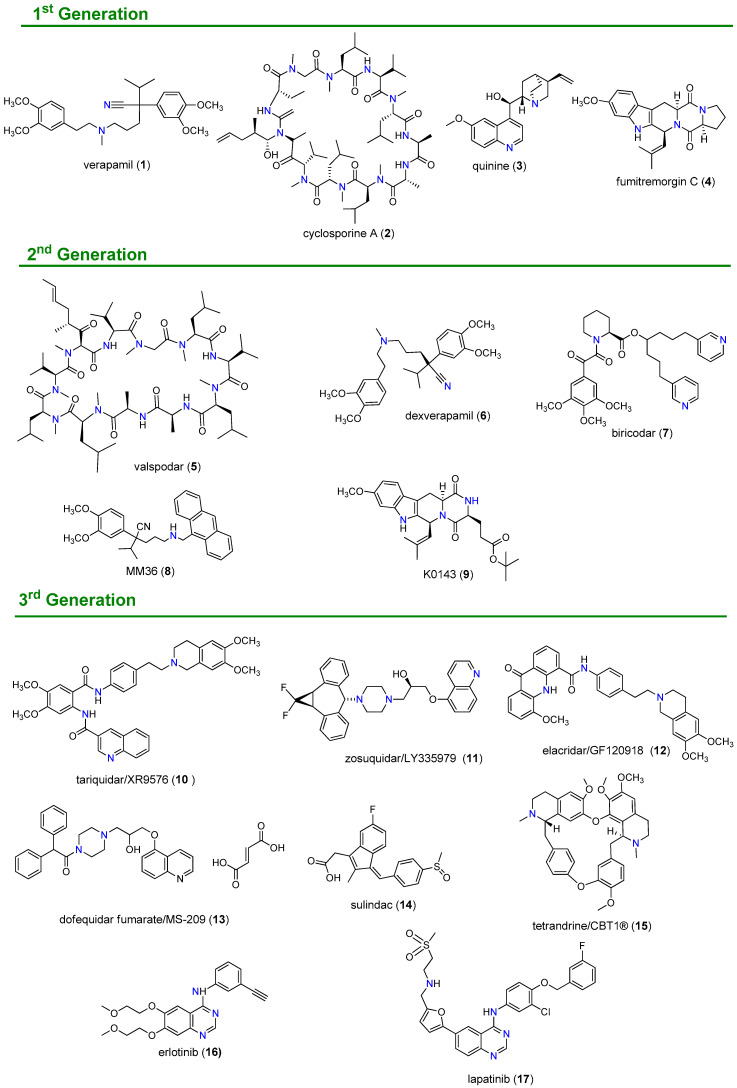
Chemical structure of selected 1st, 2nd and 3rd generation ABC transporter modulators.

Three generations of P-gp inhibitors have been developed and classified according to their specificity, toxicity and affinity (Figure 3). The first-generation comprises mostly drugs that were primarily developed for other indications but also revealed the ability to inhibit this efflux pump in cell cultures. Some of the most representative 1st generation modulators include verapamil (**1**), a calcium channel blocker, cyclosporine A (**2**), an immunosuppressant, and the antimalarial drug quinine (**3**). However, owing to their lack of specificity and low affinity for this efflux pump, high serum concentrations of these inhibitors were required, which resulted in significant toxicity levels [33]. Second-generation inhibitors, such as valspodar (**5**), dexverapamil (**6**), biricodar (**7**), and the verapamil derivative MM36 (**8**) were usually analogs of first-generation compounds designed to specifically target the efflux pump. When compared with first-generation modulators, these derivatives exhibited better affinity and specificity for this transporter, as well as reduced toxicity. However, they revealed to be substrates of drug metabolizing enzymes such as CYP 3A4 and other ABC transporters, such MRP1 and BCRP, causing significant pharmacokinetic alterations and undesirable drug-drug interactions, which hampered their clinical development [17,34,35,36]. Third-generation inhibitors, such as tariquidar (XR9576, **10**), zosuquidar (LY335979, **11**) and elacridar (GF120918, **12**), proved to have high affinity to P-gp at nanomolar range, displayed fewer pharmacokinetic interactions than inhibitors of previous generations and several were evaluated in clinical trials [33,36,37,38].

When compared with P-gp, the search for MRP1 inhibitors remains in the embryonic stage. Due to structural similarities between these two protein transporters, the first investigations on MRP1 modulators consisted in compounds previously found to modulate also the P-gp activity, such as verapamil (**1**), cyclosporin A (**3**) and biricodar (**7**), that also showed the ability to inhibit MRP1 [39]. Later, the use of rational drug design and synthetic approaches allowed the discovery of specific MRP1 inhibitors, such as dofequidar fumarate (MS-209, **13**) [40] and sulindac (**14**) [41], which reached clinical evaluation [42].

BCRP inhibitors could be divided into specific BCRP inhibitors; broad-spectrum MDR inhibitors and tyrosine kinase inhibitors (TKIs) that also inhibits BCRP, as suggested by Ahmed-Belkacem et al. [43]. The first specific BCRP inhibitor reported was fumitremorgin C (**4**), which reversed the chemo-resistance of colon carcinoma to mitoxantrone, however its neurotoxicity prevented its clinical development. Thus, K0143 (**9**) was developed from fumitremorgin C (**4**) and exhibited not only reduced neurotoxic effects but also ten times more potency in inhibiting BCRP function when compared with the parental compound [44]. The broad-spectrum of BCRP inhibitors includes compounds such as tariquidar (**10**) [45,46] and elacridar (**12**) [47], which displayed significant BCRP inhibition in addition to their potent P-gp inhibitory properties. In recent years, TKIs have been reported for their antagonist effects over P-gp, MRP1 and BCRP with encouraging results [48]. Compounds such erlotinib (**16**), and lapatinib (**17**) at high concentrations can inhibit the BCRP efflux function and so reverse MDR to chemotherapeutic drugs in cancer cells, with acceptable safety margins [49,50].

Over the years several of the above-mentioned ABC inhibitors reached clinical evaluation (Table 1), however, in most cases, the results obtained were disappointing. The success obtained was limited mainly due to unexpected side effects, toxicity from adverse interaction with anticancer drugs and poor solubility, which resulted in the premature termination of many trials [51]. Other reasons that have been mentioned by several authors for the disappointing results obtained in clinical trials were the existence of limitations and failures in the design of the studies, namely: the patients were not selected based on tumor expression of ABC transporters; in many studies the compounds evaluated were not tested at least against the three major ABC transporters; the lack of adequate preclinical models; the absence of clinically validated imaging assays to detect ABC transporter function, among others [9,31,52]. As a result, for the best of our knowledge no inhibitor of ABC transporters has been approved for clinical use so far. Therefore, there still existing an urgent need to find potent and effective MDR reversal agents with minimal adverse effects, and the solution for this quest could reside in natural products (NPs).

### 1.3. Natural Products as the Fourth Generation of ABC Transporter Inhibitors

**Figure 4:**
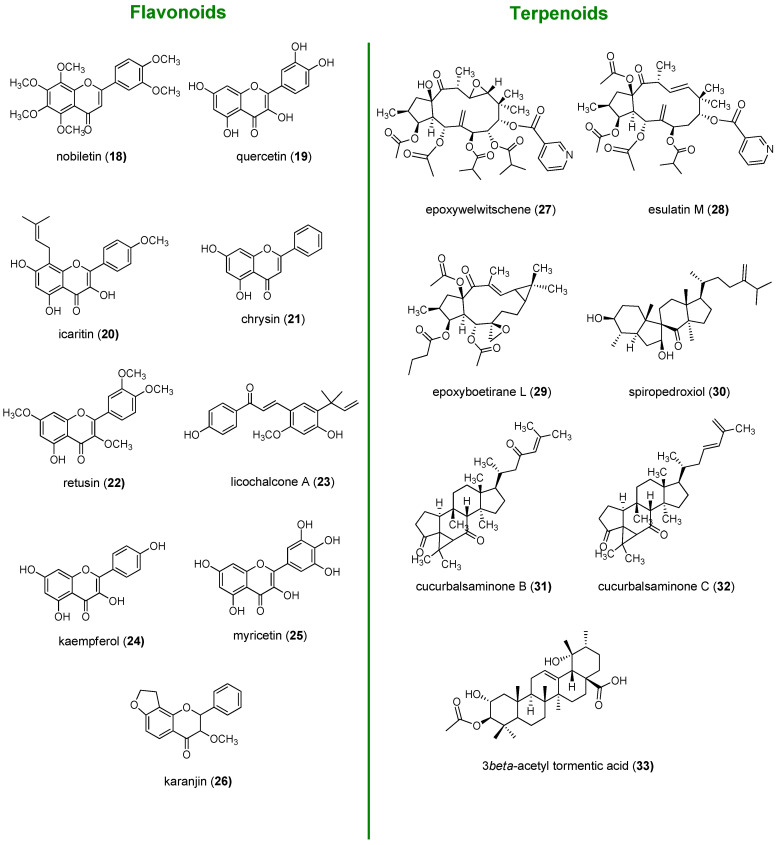
Selected naturally occurring flavonoids and terpenoids with ABC transporter modulator properties.

From a biological perspective, natural products have been evolutionary selected to bind to biological macromolecules and possess a unique chemical diversity impossible to be replicated in laboratory, which is in the origin of their broad range of biological activities. So, due to their unique characteristics, NPs have been emerged as an innovative and promising alternative for the development of efficient MDR reversers [62]. As a result of their evolutionary selection, NPs have a “promiscuous character”. This characteristic in the past was associated with dirty drugs in contrast to high-affinity single-target drugs. However, considering that MDR is a complex issue that cannot be attributed to one single mechanism of resistance, it has been proposed that the use of multifunctional drugs represent a more plausible strategy to overcome such complex phenomenon, avoiding escape mechanisms [63]. In addition, P-gp, MRP1, and BCRP are often co-expressed in several types of cancer and have an overlapped specificity for a variety of substrates. Thus, the selective inhibition of only one of the pumps was generally compensated by the remaining transporters. In these situations, broad spectrum ABC transporter inhibitors could represent a more realistic strategy to revert MDR. Thus, the ability of NPs to hit several targets, generally allied with a reduced toxicity, render these compounds attractive templates for the development of multifunctional MDR reversers [31]. A rising number of papers has been reporting the ability of NPs to act synergistically with anticancer drugs and reverse MDR in cancer cells by modulation of the three main efflux pumps P-gp, MRP1 and BCRP, being referred by some authors as the “fourth-generation inhibitors” [64,65,66]. Among NPs, flavonoids and terpenoids along with alkaloids, cardiotonic steroids and coumarins are the most extensively studied classes for their ability to inhibit these efflux proteins [63,64]. The focus of this review is the first two classes of NPs mentioned: flavonoids and terpenoids. Some of the naturally occurring flavonoid and terpenoid compounds reported for their promising MDR properties are depicted in Figure 4.

Flavonoids exert P-gp inhibitory effects through different mechanisms, including blockage of the drug binding site (nobiletin, **18** [67], Figure 4), interference with the ATP hydrolysis (quercetin, **19** [68]) and decrease of P-gp expression (icaritin, **20** [69]). Flavones, such as chrysin (**21**) and retusin (**22**) as well as the chalcone licochalcone A (**23**), were found to be among the most promising BCRP modulators due to their high inhibitory potencies and low toxicities [70,71,72], whereas kaempferol (**24**) and myricetin (**25**) displayed potent MRP1 inhibition [73]. Some flavonoids, such karanjin (**26**), have the ability to act as broad spectrum ABC inhibitors. It was reported that karanjin (**26**) was able to interfere with ATPase activity and drug efflux mediated by all three transporters, P-gp, MRP1, and BCRP [74].

Terpenoids, namely diterpenes and triterpenes, have been widely reported for their ability to inhibit ABC transporters and reverse MDR [75,76,77,78]. An extensive research on *Euphorbia* species allowed the identification of macrocyclic diterpenes of the jatrophane and lathyrane-type as strong P-gp modulators, displaying as well synergistic interactions with cytotoxic drugs, such as the jatrophanes epoxywelwitschene (**27**, Figure 4) and esulatin M (**28**) [79,80], and the lathyrane epoxyboetirane L (**29**) [81]. The tetracyclic triterpenoid spiropedroxodiol, with a *spiro* scaffold (**30**), isolated from *Euphorbia pedroi*, was also found to be strong P-gp modulator [82]. Triterpenoids such as cucurbalsaminones A (**31**) and C (**32**), featuring a unique 5/6/3/6/5-fused pentacyclic carbon skeleton, displayed strong inhibition of P-gp-mediated transport [78]. Another triterpene, 3β-acetyl tormentic acid (**33**), exhibited potent ABCC subfamily efflux function modulation with greater selectivity for MRP1 [83].

Despite the promising anti-MDR properties of natural flavonoids and terpenoids, they need to be optimized to have the desired potency, selectivity, and pharmacokinetic properties of a clinically useful drug. Optimization of the basic scaffold to improve these properties can be accomplished though preparation of semisynthetic derivatives.

### 1.4. Nitrogen-Bearing Compounds as Modulators of ABC Transporters

The presence of a basic nitrogen atom has for a long time been recognized as a relevant structural requirement of ABC modulators, particularly of P-gp. This relevance is also supported by the analysis of Figure 3, where the majority of 1st, 2nd and 3rd generations of MDR reversers share as common feature the presence of at least one basic nitrogen [84]. The first structure-activity relationship (SAR) studies on verapamil (**1**) identified two key structural features: a basic linker located between two aromatic residues [85]. Similar results were obtained in SAR studies performed with a series of analogues of reserpine, which identified the lipid solubility at physiological pH, the spatial disposition of two aromatic planar rings and the presence of one basic nitrogen atom as key features for the P-gp modulatory activity [86].

**Figure 5:**
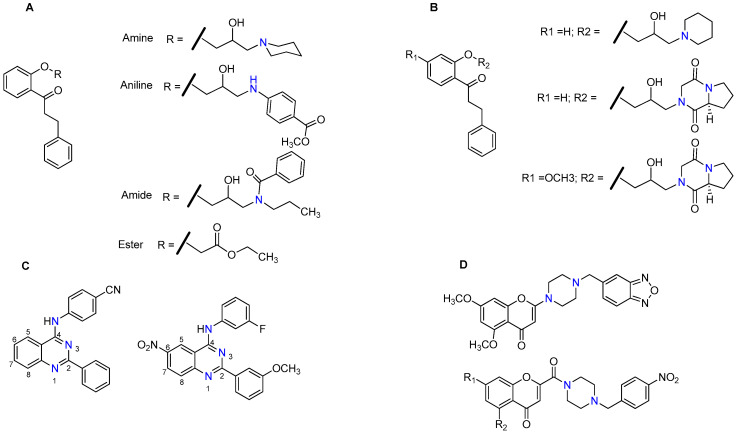
Examples of propafenone (**A**) prepared by Heckler et.al. [87] and (**B**) prepared by Cramer et al. [88]) quinazolide (**C**) and chromone (**D**) derivatives bearing nitrogen substituents.

In 1999, Heckler and co-workers, using a set of propafenone derivatives bearing tertiary amine, aniline, amide and an ester moieties (Figure 5A), reported that the presence of a basic nitrogen atom is not an absolute requirement for the activity since all compounds, including the ester derivative, which lacks a nitrogen atom, exhibited P-gp-inhibitory potential. However, they found an excellent correlation between the hydrogen bond acceptor strength and the P-gp inhibitory potency, suggesting that the interaction of nitrogen with P-gp is determined by its strong electron donor capability, which confers a beneficial effect for the inhibitory activity [87]. Similarly, one year later, Seelig et al. found that if two substrates interact simultaneously with P-gp, the compound with the highest potential to form hydrogen bonds generally behaves as a competitive P-gp inhibitor [19]. Later, in 2007, Cramer et al., using a series of propafenone derivatives modified in the vicinity of the nitrogen atom (Figure 5B), observed that the selectivity towards P-gp was defined by hydrogen bond strength of the nitrogen atom. This feature apparently was not essential for the activity towards BCRP [88]. The same research group extended these studies with the propafenone scaffold by searching for the molecular features that trigger P-gp *vs.* BCRP, selectivity. They found that the selectivity towards P-gp was influenced by both basicity of the nitrogen atom and the flexibility of its substituents, whereas the selectivity towards BCRP was apparently more related with the substituent on the central aromatic ring than with the basicity of nitrogen atom [89]. However, recently, in a series of modifications of the quinazoline scaffold, Wise’s group found that in addition to an substituted aromatic residue at position 2, the presence of a substituted aniline linker at position 4, a nitro function at position 6 and mainly a nitrogen atom at position 3 (Figure 5C) were crucial for activity and selectivity toward BCRP [90,91]. Other reports have been suggesting a set of characteristic features of BCRP inhibitors, which include: the lipophilicity, the planar structure and an amine bounded to one carbon of a heterocyclic ring [44].

Regarding MRP1, a recent study revealed that chromone derivatives, bearing amino groups or *N*-substituted carboxamine functional groups (Figure 5D), displayed potent and selective inhibition of this efflux pump [92].

The great majority of naturally occurring flavonoids and terpenoids do not have nitrogen atoms and, in the case of terpenoids, the aromatic moieties are frequently absent. Thus, one of the strategies that has been adopted by several research groups for optimizing their structures as ABC transporter modulators is the introduction of nitrogen-bearing moieties into the flavonoid and terpenoid backbone, many times combined with aromatic substituents. This review covers recent findings on nitrogen-bearing flavonoid and terpenoid derivatives and their ability to inhibit ABC efflux transporters and revert MDR. Thus, SAR considerations on the influence of the introduction of nitrogen bearing groups for the MDR reversal properties of the new compounds generated will be highlighted. Despite the existence of other published review articles that put emphasis on the potential of flavonoids and terpenoids as MDR reversal agents in cancer, as long as we know, this is the first review that focus on the influence of nitrogen introduction on their ABC modulatory properties.

## 2. Flavonoids

**Figure 6:**
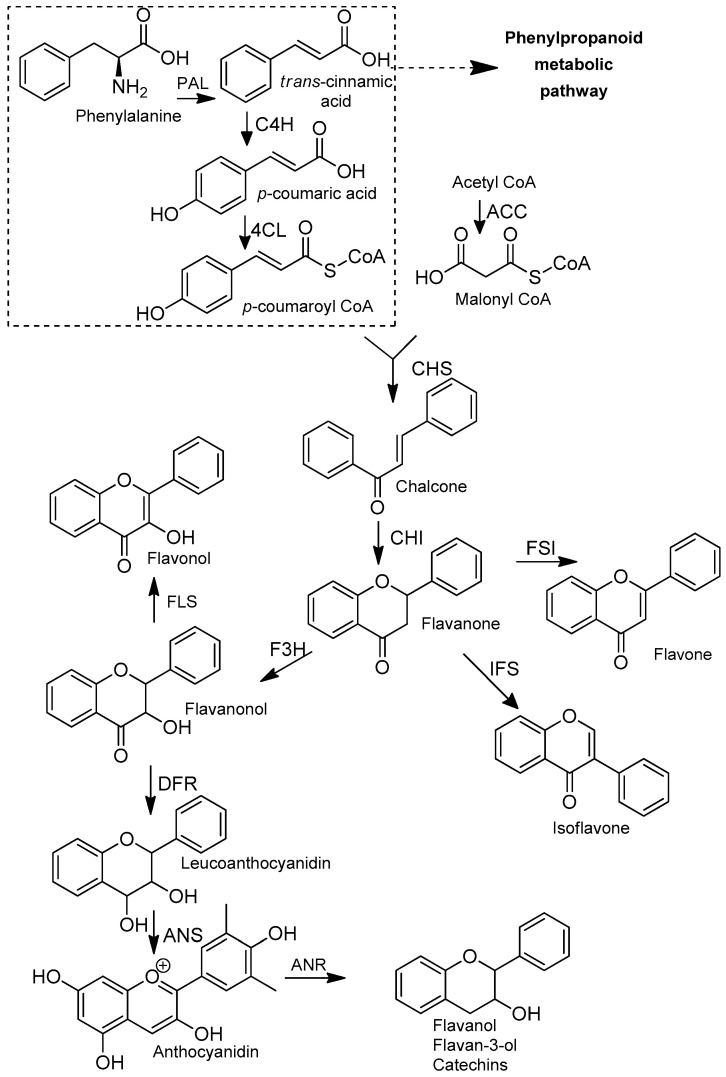
Biosynthetic pathway of flavonoid scaffolds. PAL: phenylalanine ammonia-lyase; C4H: cinnamate 4-hydroxylase; 4CL: 4-coumaric acid: CoA ligase; ACC: acetyl-CoA carboxylase; CHS: chalcone synthase; CHI: chalcone isomerase; F3H: flavanone 3-hydroxylase; FLS: flavonol synthase; DFR: dihydroflavonol 4-reductase; ANS: anthocyanidin synthase; ANR: anthocyanidin reductase; IFS: 2-hydroxyisoflavone; FSI: flavanone synthase [95].

Flavonoids are secondary metabolites belonging to the class of polyphenols. These metabolites are ubiquitously distributed in the plant kingdom, and widely present in several medicinal plants and in human diet. Flavonoids have a great therapeutic value as they are relatively potent antioxidants and chemo-protectants, generally with low or negligible toxicity [93,94]. Most of the flavonoids already identified are biosynthesized via phenylpropanoid metabolic pathway based on the transformation of the phenylalanine into *p*-coumaroyl CoA (Figure 6) [95].

**Figure 7:**
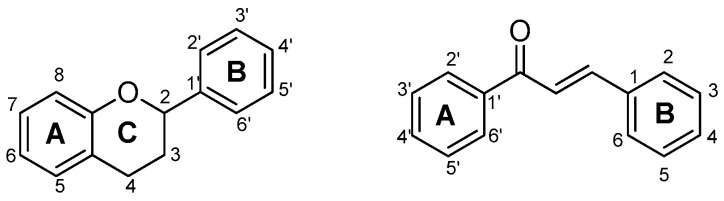
Flavonoids general scaffold with the carbons numbered.

The flavonoid scaffold, more precisely the chalcone unit, is obtained after combining *p*-coumaroyl-CoA with three malonyl-CoA molecules via Claisen-type condensation. Thereafter, different flavonoid subclasses can be obtained depending on the enzymes (isomerases, reductases, hydroxylases and dioxygenases) that modify the main skeleton, accordingly. The general flavonoid structure contains a 15-carbon skeleton constituted by two benzene rings (ring A and ring B) which are connected by a heterocyclic ring containing an oxygen (ring C) (Figure 7). The subclasses can be classified, based on the unsaturation and oxidation of ring C or the carbon in which the ring B is attached to ring C, in: chalcones, flavones, isoflavones, flavonols, flavanones, flavanonols, leucoanthocyanidins and flavanols (Figure 6) [95].

The reader is advised to consider the representations of the flavonoid general scaffold with the carbons numbered depicted in Figure 7, for a better understanding of the structural modifications discussed in the following sections.

It has been exhaustively reported that flavonoids can contribute positively to efflux pump inhibition, mostly when owning a hydroxyl group at position 5, a methoxy group attached to carbon 3 and/or double bond between carbons 2 and 3 [96]. However, no overviews of nitrogen-containing flavonoids exhibiting potential to effectively modulate P-gp, MRP1 and BCRP activities have been published so far. So, the next few sections will describe several semisynthetic nitrogen-bearing flavonoid derivatives, which were reported as MDR reversers.

### 2.1. Chalcones

**Figure 8:**
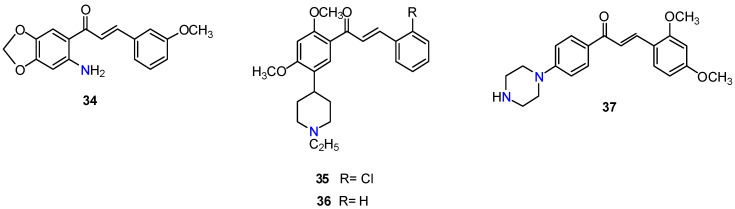
Chalcone derivatives **34**–**37**.

The structural modification of rings A and B of chalcone scaffold has been a frequently explored strategy to produce derivatives with improved ABC transporters inhibitory activities. One of the first reports on nitrogen bearing-flavonoid derivatives dates to 2000, when Xia and co-workers prepared a series of 6′-amino chalcone derivatives that exhibited significant activity toward human epidermoid carcinoma of the nasopharynx (KB-VIN) cell line, overexpressing P-gp. The results were expressed as IC_50_ values. IC_50_ is defined as the concentration of compound that produced 50% P-gp inhibition, thus lower IC_50_ values mean stronger P-gp inhibitory properties. The most active compound, **34** (Figure 8), displayed an IC_50_ value of 1 µM [97]. Later studies revealed that methoxylated chalcones with basic functionalities displayed better P-gp inhibitory activity than their non-basic counterparts. Two of the most active derivatives, **35** and **36** (Figure 8), possess a 5-(1-ethylpiperidin-4-yl) group at A-ring and strongly blocked the P-gp efflux of calcein-AM and doxorubicin in P-gp overexpressing MDR MDCKII/MDR1 and MCF-7/Adr cells to a greater degree than verapamil (**1**). SAR studies revealed that the introduction of basic groups, preferably in the A-ring, was necessary but not enough for the P-gp inhibition, since the presence *meta*-dimethoxy motifs on either ring A or B was also essential for the activity. In contrast to their marked effects on P-gp efflux activity, these basic-chalcone derivatives exhibited negligible activity when screened for BCRP inhibition. Interestingly, derivative **37**, bearing a piperazin moiety at A-ring, inhibited both P-gp and BCRP, to a similar extent as verapamil (**1**) and fumitremorgin C (**4**), respectively, without presenting significant cytotoxicity. Thus, compound **37** may be a useful template for the design of dual P-gp/BCRP inhibitors [98].

More recently, the replacement of chalcone B-ring with a quinoxaline moiety combined with different patterns of hydroxy and methoxy substitution at A-ring resulted in a series of derivatives (e.g., compounds **38**–**40**, Table 2) with significant BCRP inhibitory effects in HEK293-ABCG2 cells. The most active quinoxaline derivatives (**38**–**40**) displayed IC_50_ values within the range of 1.4–1.9 µM (Table 2). Further studies revealed that quinoxaline-substituted chalcones consistently exhibited higher BCRP inhibitory effect than the corresponding B-ring analogues 2-naphthyl (compound **38** *vs.* **41** and **39** *vs.* **42**) or 3,4-methylenedioxyphenyl (compound **38** *vs.* **43** and **39** *vs.* **44**). SAR analysis also suggested the need of two or three methoxy groups attached to the phenyl A-ring to produce a maximal inhibition. The positive contribution of the quinoxaline moiety at B-ring for the activity of the compounds was apparently related to electrostatic interactions of the two nitrogen heteroatoms with the efflux protein [99].

**Figure 9:**
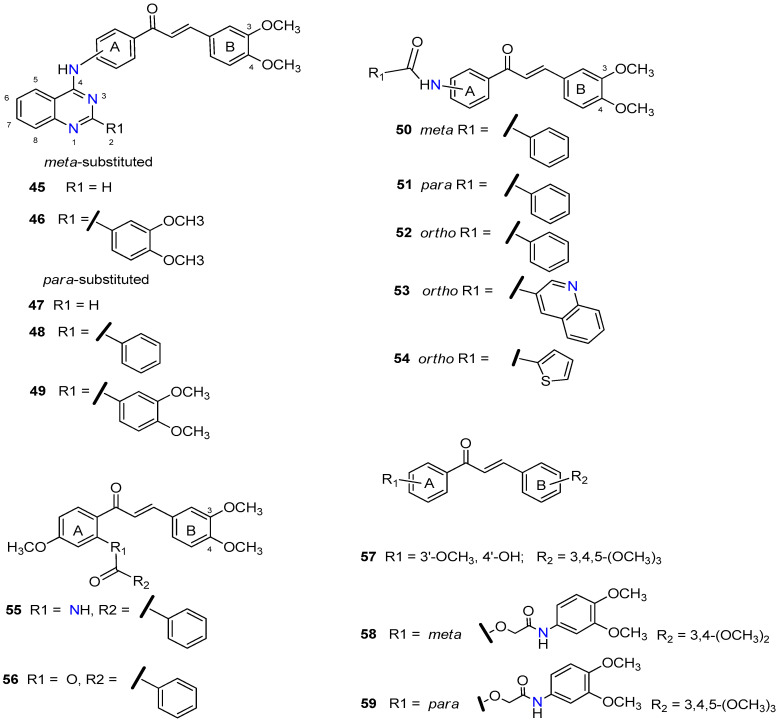
Chalcone derivatives **45**–**59**.

The promising BCRP inhibitory activity of chalcones was further explored by Wiese and co-workers by linking a quinazoline ring to the A-ring of chalcone giving origin to a series of *meta*-acryloyl (e.g., **45** and **46**, Figure 9) and *para*-acryloyl (e.g., **47**–**49**, Figure 9) heterodimeric derivatives [100]. The maximum BCRP inhibitory activities were obtained with the *para*-substituted compounds with a 3,4-dimethoxyphenyl substituent at position 2 of quinazoline and a 3,4-dimethoxy substitution at B-ring of chalcone, with **49** being the most active compound of this series (IC_50_ = 0.19 µM, Table 3) in MDCK II BCRP cells. This derivative was not only able to reverse the MDR towards SN-38 at the same concentration range than the standard BCRP inhibitor Ko143 (**9**), but also presented reduced toxicity (GI_50_ = 92.90 µM, GI_50_ is defined as the concentration of compound that inhibited cell growth by 50%, used to access intrinsic cytotoxicity of tested compounds), which is important for a high therapeutic ratio [100]. In addition, enzyme kinetic studies revealed that compound **49** behaves as a non-competitive inhibitor of BCRP and was not transported by the efflux pump. This compound exhibited only a weak inhibition of P-gp function (IC_50_ = 14.9 µM, Table 3), therefore its ability to reverse MDR and is nearly selective toward BCRP. Differently, the meta-substituted compounds **45** and **46** showed substantial P-gp inhibitory activity (IC_50_ = 0.42 µM and 0.48 µM, respectively, when tested in A2780adr cell line). Interestingly, compound **46** had similar BCRP and P-gp inhibition (IC_50_ = 0.60 µM *vs.* 0.48 µM, respectively) activities, which renders this compound a promising template for the development of a dual P-gp/BCRP inhibitor [100].

The introduction of differently substituted amides at positions 2′, 3′, and 4′ on chalcone A-ring originated a series of acryloylphenylcarboxamides derivatives (e.g., **50**–**54**, Figure 9) [101]. Some derivatives inhibited the efflux of both BCRP substrates, pheophorbide A and Hoechst 33342, in BCRP overexpressing MDCK II BCRP cell line, which indicated that their inhibitory activity was independent of substrate. For example, compound **52**, bearing an *ortho*-benzamide moiety, displayed significantly higher inhibitory activity (IC_50_ = 0.98 µM in the pheophorbide assay, Table 3) than the corresponding *meta*-substituted (**50**) (IC_50_ = 2.18 µM) and *para*-substituted (**51**) (IC_50_ = 1.30 µM) analogues [101].

In addition, the replacement of the phenyl ring of compound **52** by heterocyclic moieties, gave rise to the most active BCRP inhibitor of this series: the 2-thienyl derivative **54** (IC_50_ = 0.60 µM) [101]. Further modifications showed that the introduction of the 4′-methoxy group at A-ring further increased of the BCRP inhibitory activity, as observed by comparing **55** (IC_50_ = 0.22 µM, Hoechst 33342 assay) [102] with its analogous compound **52** (IC_50_ = 0.50 µM) [101].

On the other hand, the replacement of the A-ring amide linker with an ester group markedly decreased the BCRP inhibition properties (compare IC_50_ **55** *vs.* IC_50_ **56**, Table 3), which support the beneficial effect of the amide linker for the inhibitory activity of the chalcones. These overall results depicted four key structural features that favors the BCRP inhibition, namely: the *ortho*-position of the amide linker; 3,4-dimethoxy substitution on ring B; and the presence of a phenyl or 2-thienyl substitution at the amide linker. The selectivity of all derivatives was assessed by evaluating their effects on P-gp-overexpressing A2780 adr and the MRP1-overexpressing H69 AR cell lines [101,102]. The majority of derivatives displayed no MRP1 inhibition and only a reduced affinity toward P-gp was observed. Differently, compound **53** displayed relevant BCRP and P-gp inhibition with IC_50_ values of 0.57 and 0.49 µM, respectively, thus, this compound could act as dual BCRP/P-gp inhibitor. The reduced toxicity exhibited by the promising derivatives **53**, **54**, **55** (GI_50_ = 65.90 µM, 93.20 µM, 78.00 µM respectively) toward MDCK II BCRP cells, renders these derivatives a high therapeutic ratio [101,102].

Recently, a panel of chalcone derivatives with varying substitutions on the A and B rings (e.g., derivatives **57**–**59,** Figure 9) was prepared and evaluated for their inhibitory potential towards BCRP, P-gp and MRP1 in pheophorbide A (BCRP) and calcein AM (P-gp and MRP1) assays (Table 3) [103]. Derivative **57**, which does not have any nitrogen in its structure, was the most active BCRP inhibitor (IC_50_ = 1.97 µM). However, no P-gp or MRP1 inhibition was displayed by this compound that showed significant intrinsic toxicity, with GI_50_ values in the single-digit micromolar concentration range, rendering it a reduced therapeutic ratio. The *meta*-dimethoxyphenyl amide at the A ring combined with a 3,4-dimethoxy substituent at the B-ring originated compound **58** which displayed only moderated BCRP inhibitory activity (IC_50_ = 6.33 µM). On the other hand, the *para* substituted derivative **59** displayed not only a higher BCRP inhibition (IC_50_ = 3.37 µM), but also exhibited inhibitory potential against MRP1 (IC_50_ = 12.5 µM, in ABCC1- overexpressing H69AR cell line). Noteworthy, compound **59** sensitized BCRP-overexpressing MDCK II BCRP cells with regard to SN-38 with an EC_50_ value of 0.107 µM, which can be compared to highly potent MDR reversing agents such as ceritinib (EC_50_ = 0.106 µM) or linsitinib (EC_50_ = 0.282 µM). EC_50_ is defined as half-maximal MDR reversal concentrations and represent the concentration of derivative needed to reduce the GI_50_ of the corresponding antineoplastic agent (SN-38) by 50%. Lower EC_50_ values mean stronger MDR reversal properties values. Thus, the results of this study suggested that the *para*-substituted amide derivative **59** is a highly potent reverser of BCRP-mediated MDR and displays BCRP/MRP1 dual inhibition [103].

Yin and co-workers prepared a series of derivatives by introducing a heterocyclic ring bearing basic nitrogen at chalone B-ring (ex. **60**–**64**, Table 4) and evaluated their chemo-sensitizing effect in MCF-7/DOX cells [84]. Reverse fold (RF) was used as a measure of the potency of the tested compounds as MDR reversers (Table 4**)**. Compounds **61** and **64,** bearing a 3-piperazinyl substituent, showed the highest activity (RF = 50.19 and 19.46, respectively) in reversing doxorubicin (DOX) resistance. By comparison with **61,** which has a free *N*-terminal piperazine group, compounds **60** and **63,** possessing 1-methyl-piperazinyl and 3-morpholinyl substituents, respectively, exhibited a significant decrease of the chemo sensitizing effect (RF = 2.17 and 1.62, respectively). These results suggest that the introduction of groups at C-3 of chalcone B-ring, which can form strong hydrogen bonds with P-gp (like the piperazinyl moiety without substitutions at the terminal nitrogen atom), substantially improve the MDR-reversal effects. Differently, the substitutions at C-4 on ring B showed no positive effect on reversing P-gp-mediated DOX-resistance (compare RF values of **61** *vs.* **62**, Table 4). Further studies revealed that compound **61** exhibited high selectivity for P-gp protein over BCRP or MRP1. In addition, this compound reversed P-gp—mediated MDR in nude mice bearing MCF-7/DOX tumor xenografts by increasing the efficacy of doxorubicin [84].

### 2.2. Flavanones and Flavanonols

Recently, a study regarding derivatives of the flavanone naringenin (**65**) as inhibitors of P-gp, MRP1, and BCRP was performed by Ferreira et al. [104]. The carbonyl group was modified into a series of derivatives containing nitrogen atoms and extra aromatic moieties such as hydrazones, azines and carbohydrazide derivatives aiming at increasing their inhibitory properties (e.g., **66**–**69**, Table 5). The ability of naringenin derivatives to modulate P-gp, MRP1 and BCRP was evaluated in overexpressing P-gp (NIH/3T3), MRP1 (BHK-21) and BCRP (HEK-293) cell lines. The results were presented as percentage of transporter inhibition, which was accessed by comparison with the 100% of inhibition obtained with verapamil (35 μM, MRP1), elacridar (5 μM, P-gp) and Ko143 (1 μM, BCRP). None of the derivatives evaluated proved to be active in inhibiting P-gp. However, the carbohydrazides **66** and **67** showed potent MRP1 efflux inhibition with an extension of 62.6% and 95.0%, respectively. Based on SAR analysis, the authors suggested that the spatial orientation of the aromatic substituent of the carbohydrazide derivatives was directly correlated with the activity due to an ‘out-of-plane’ torsion that maximize interactions with the efflux pump. Additionally, the azine derivatives **68** and **69** were found to display an interesting inhibitory activity pattern towards both MRP1 (efflux inhibition of 56.3 and 45.1%, respectively) and BCRP (efflux inhibition of 53.5 and 71.1%, respectively) pumps, rendering these compounds the ability to act as dual MRP1/BCRP inhibitors. These inhibitory results for azine derivatives **68** and **69** prove that the introduction of the C=N-N=C chain could be important for the ability of these compounds to inhibit both BCRP and MRP1 pumps [104].

**Figure 10:**
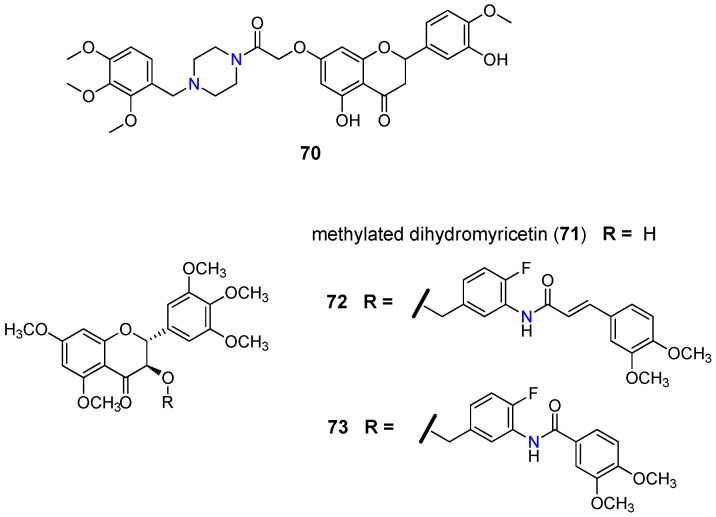
Structure of 2,3,4-trimethoxybenzylpiperazine flavanone **70**, methylated dihydromyricetin (**71**) and its derivatives **72** and **73** [107].

Schwaller and co-workers found that 7-(*N*-benzylpiperazinyl) flavanones displayed better reversal activities than verapamil (**1**) against P-gp [105]. Their research revealed that the 2,3,4-trimethoxybenzylpiperazine derivative **70** (Figure 10) displayed the best results, suggesting that the increase of the basicity of piperazine nitrogen atoms, caused by electron-donating effects, together with the lipophilic character may influence the modulatory activity [106].

Regarding the flavanonols subgroup, Wong et al. [107] reported a library of seventy methylated dihydromyricetin (**71**) derivatives (e.g., **72** and **73**, Figure 10) showing reversal activity towards P-gp. The modulating performance was measured by reversal fold (RF) defined as a ratio between GI_50_ of paclitaxel in LCC6MDR cells in the absence and presence of 1 µM of the modulator. Derivatives containing only two methoxy groups, together with the rigid conformation attributed to the oxycarbonylphenylcarbamoylvinyl linker (**72**) and oxycarbonylphenylcarbamoyl (**73**) bearing nitrogen atoms, gave the best inhibitory results for P-gp, with a RF of 10 and 15.4, respectively, compared to the 3.8 obtained for verapamil (**1**) [107].

### 2.3. Flavones

**Figure 11:**
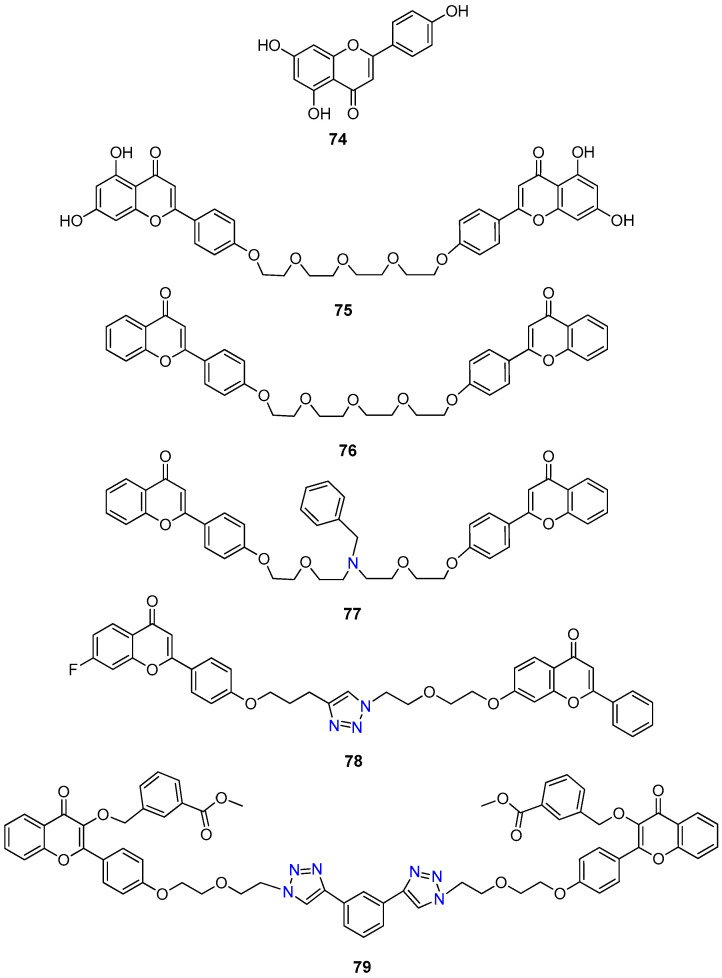
Chemical structures of apigenin (**74**) and flavone dimer derivatives **75**–**79.**

The synthesis of flavone dimers has been emerging as an interesting strategy to improve their MDR-reversal properties. This strategy is supported by the increased affinities and specificities displayed by polyvalent ligands towards their biological targets when compared to monovalent ligands [108]. On the other hand, many ABC transporters possess a pseudodimeric structure, which also supports a bivalent approach. Chow and co-workers have been developing extensive research in the synthesis of flavonoid dimers. The first studies evaluated the ability of apigenin (**74**) homodimers (Figure 11) to modulate the P-gp function by determining the half-maximal MDR reversal concentrations (EC_50_, Table 6).

EC_50_ is defined as the concentration of flavonoid dimer needed to reduce the GI_50_ of determined anticancer agent against MDR cells by 50%, thus, lower EC_50_ values mean stronger MDR reversal properties. Results revealed that derivatives bearing a four units polyethylene glycol (PEG) linker, such as compound **75** (EC_50_ = 0.95 µM) and its dehydroxylated derivative **76** (EC_50_ = 0.22 µM), displayed significantly higher potency to modulate P-gp-mediated paclitaxel resistance than the monomeric apigenin (**74**) in LCCMDR cell line. Later, a great improvement in reversal potency was achieved by introducing an amine group into the PEG linker obtaining derivative **77** bearing a *N*-benzyl group (EC_50_ = 0.15 µM) [108,111]. This compound, in nanomolar range (EC_50_ ranging from 0.09 to 0.179 µM), was able to increase the chemosensitivity of LCC6MDR cells to several anticancer drugs such paclitaxel, vinblastine, vincristine, doxorubicin, daunorubicin, and mitoxantrone to levels of parental LCC6 cells. In fact, derivative **77** was found to be 11- to 46-fold more potent than first generation inhibitor verapamil (**1**). Its MDR reversal activity correlated with the inhibition of P-gp efflux function by binding to the substrate-binding pocket [108,111]. Further studies revealed that at 0.5 µM, compound **77** displayed preferential inhibition of P-gp over other ABC transporters, since it showed only weak modulating activity towards MRP1 and BCRP in 2008/MRP1 and HEK293/R2 cells, respectively. Importantly, **77** presented other advantageous properties including reduced toxicity (GI_50_ = 85.0 μM for L929 cells), high therapeutic index (574.3) and improved water solubility [111]. The low toxicity of derivative **77** was confirmed in vivo since no significant body weight reduction or animal deaths were observed after Balb/c mice treatment with **77** at 90 mg/kg together with paclitaxel at 12 mg/Kg i.p. [108].

Copper(I) catalyzed Huisgen 1,3-dipolar cycloaddition conditions were used to prepare a vast panel of triazole bridged flavonoid homo- and heterodimer derivatives that induced relevant MRP1 inhibition (EC_50_ ranging from 0.053 to 0.298 µM) and presented reduced toxicity, thus having high selective indexes ranging from 190 to 1887. Within these, compound **78** was the most active in reversing doxorubicin resistance (EC_50_ = 0.053 µM, Table 6). Some of the structural features that revealed to have beneficiary effect for MRP1 modulation, include the dimeric structure, phenyl unsubstituted A and C flavone rings, an ideal linker size between 13 and 17 atoms and the presence of the triazole ring within the linker [112]. It should be highlighted that due to the presence of the basic triazole ring, derivative **78** exhibited good water solubility, an important characteristic for further in vivo studies. Docking studies suggested that compound **78** binds to the bipartide binding site of MRP1 with stronger affinity than doxorubicin, a known MRP1 subtract, working as a competitive inhibitor. Additionally, derivative **78** at 1 µM revealed ability to potently reverse the P-gp-mediated paclitaxel resistance in LCC6MDR cells (RF = 40.7) and BCRP-mediated topotecan resistance in HEK293/R2 cells (RF = 18.2), which renders this compound as a promising lead to a rare MRP1/BCRP/P-gp inhibition [112].

Recently, a new series of mono- and bistriazole-linked flavonoid dimers was evaluated for their modulator activity towards BCRP, P-gp and MRP1 [113]. Despite most of the derivatives did not show any promising activity toward MRP1 and P-gp, several derivatives presented interesting BCRP-modulating activity on HEK293/R2 and MCF7-MX100 cells. The presence of the two flavonoid moieties, the linker length and a benzyloxy substitution at position 3 of the flavone C-ring were considered key structural features for the high BCRP inhibitory activity. Derivative **79**, bearing a *m*-methoxycarbonylbenzyloxy substitution at C-3 of the flavone moieties and a bis-triazole-containing linker (21 atoms), displayed a higher BCRP inhibitory activity than Ko143 (**9**) (EC_50_ (**79**) = 0.001 and 0.002 µM *vs.* EC_50_ (Ko143, **9**) = 0.009 and 0.009 µM, Table 6) towards both HEK293/R2 and MCF7-MX100 cells. The high selectivity of **79** for the BCRP is likely related with the length of the linker between the two flavonoid moieties (13–27 atoms). Differently, P-gp and MRP1 inhibitors required an optimal linker length between the two flavones with about 13−15 atoms and 13−17 atoms, respectively [113]. Derivative **79** was also found to inhibit the BCRP-ATPase activity and block BCRP drug efflux function restoring the mitoxantrone and topotecan sensitivity. In silico docking studies suggested that this derivative bind preferentially to BCRP at DBS and blocks the access for substrates. The linker 1,3-bis-(triazol-4-yl)-benzene was apparently responsible for the strong interaction with the protein, through π-π interactions with residues Phe439 of helix 2′ and Phe547 of helix 5a. [113].

A set of new “hybrid” aminoester heterodimers, carrying an [(*E*)-3-(3,4,5-trimethoxy-cinnamoyl)] residue connected to a flavone or chromone moiety through an *N*-containing linker, was synthesized (e.g., derivatives **80**–**84**, Table 7) [114]. P-gp inhibitory activity, evaluated on human leukemia K562/DOX doxorubicin-resistant cells, was expressed by [I]_0.5_ and α_max_ (Table 7). [I]_0.5_represents the concentration that causes a half-maximal increase in the nuclear concentration of pirarubicin and measures the potency of the modulator. α_max_-represents the maximum increase in the nuclear concentration of pirarubicin in resistant cells, and its value varies between 0 (in the absence of the inhibitor) and 1 (when the amount of pirarubicin in resistant cells is the same as in sensitive cells). According to the results obtained, these derivatives displayed remarkable efficacies since they completely reversed P-gp-dependent pirarubicin extrusion (α_max_ close to 1), with potency values in the micromolar range. The polymethylenic derivatives (e.g., **81**, Table 7) were always more active than ethoxyethylic analogs (e.g., **84**, Table 7) and the introduction of a methyl group or a *N*-benzyl substitution on the basic nitrogen increased the activity (compare results for **81** *vs.* **83** and **82** *vs.* **83**, Table 7) [114]. The nature of the linker proved to be determinant for the P-gp modulatory activity due to its critical interactions with the pump. Compound **81** ([I]_0.5_ = 0.34 µM), bearing a lipophilic non-substituted flavone moiety, showed the highest reversing activity, being 5-fold more active than verapamil (**1**) ([I]_0.5_ = 1.6 µM) [114].

Modifications at position 3 of the flavone core are among the most commonly reported in order to produce novel flavone-based ABC modulators. A panel of C-3 flavone derivatives, bearing an acetamido linker, was synthesized and evaluated for their inhibitory potential against P-gp, MRP1, and BCRP [103]. While the isobenzofuranone- and phenyl-substituted flavone derivatives did not exhibit any inhibitory activity against ABC protein transporters, different results were obtained for the naphtyl-substituted flavone derivatives **85**–**87** (Table 8). Derivative **85** was found to be a potent and selective P-gp inhibitor with an IC_50_ value of 1.98 µM, allied with a relatively safe profile with a therapeutic ratio of over 50. Compound **86** was also a potent P-gp inhibitor (IC_50_ = 1.41µM), however it reached only 50% of the maximum inhibition level, in comparison with cyclosporine A (**2**). The other naphthyl derivative (**87**) showed moderated inhibition against P-gp (IC_50_ = 5.43 µM) and a good inhibition of BCRP (IC_50_ = 3.75 µM), having a promising potential for the development of a dual P-gp/BCRP MDR modulator [103].

### 2.4. Flavonols

Among flavonols, quercetin (**19**) is receiving a great deal of attention due to its several therapeutic properties, such as anticancer, antioxidant, anti-inflammatory, antiapoptotic, antidiabetic, antiobesity, among others [115,116,117]. Quercetin-aminoacid conjugates linked to 7-*O* and/or 3-*O* positions were also reported as promising P-gp modulators [118]. As tested by Kim et al., the conjugates **88** and **89**, with alanine and glutamic acid substituents at the 7-*O* position through an amide linker, were the most effective in reversing the P-gp-mediated doxorubicin-resistance in MES-SA/Dx5 cell line (Table 9). In particular, the derivative **89** had the best inhibition performance with an IC_50_ value of 0.14 µM, which corresponds to 30.5-fold increase in MDR-reversal activity when compared to quercetin (**19**). Compounds **90** and **91,** with 3-*O* aminoacid substituents, also showed promising P-gp inhibitory activity. The authors proposed that a combination of better stability, solubility and cellular uptake by implementing flavonoid-amino acid conjugates is a plausible reasoning on what lies behind the enhancement of the MDR-modulating activity [118].

### 2.5. Flavanols

**Figure 12:**
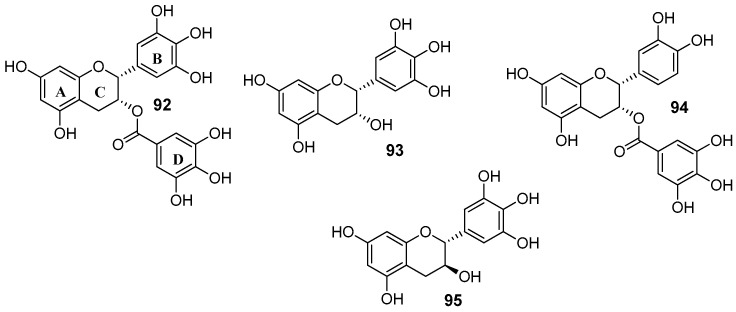
Structures of catechins: (−)-epigallocatechin gallate (**92**), (−)-epigallocatechin (**93**), (−)-epicatechin gallate (**94**) and (+)-gallocatechin (**95**).

In the set of flavanols, epigallocatechin-3-gallate (**92**) is the active compound of green tea and represent around 30% of dry tea leaves [119]. Beyond this most abundant catechin in green tea, other polyphenols can be found in this beverage such as epigallocatechin (**93**), epicatechin gallate (**94**) and gallocatechin (**95**) (Figure 12) [120].

Wong et al. reported a library of methylated epigallocatechin (**93**) and gallocatechin (**95**) derivatives, showing reversal activity towards P-gp. The modulating performance was measured by RF (Table 10) [107]. Among the epigallocatechin (**93**) derivatives, it was observed that the introduction of a rigid oxycarbonylphenylcarbamoyl linker at C-3 gave rise to compounds **96** and **97**, which displayed 10- and 11.5-times higher P-gp modulatory activity than verapamil (**1**), respectively. The same logical reasoning was applied to gallocatechin (**95**) derivatives and the best results obtained were once again for the compounds containing the same rigid linker, **98** and **99**. These derivatives inhibited P-gp drug efflux with around 8- and 14-fold more potency than verapamil (**1**), respectively. In addition, derivatives **98** and **99** showed good modulation activity against BCRP (RF = 10.4 and 12.3, respectively) and moderate activity against MRP1 (RF = 2.6 for both compounds) [107].

## 3. Terpenes

**Figure 13:**
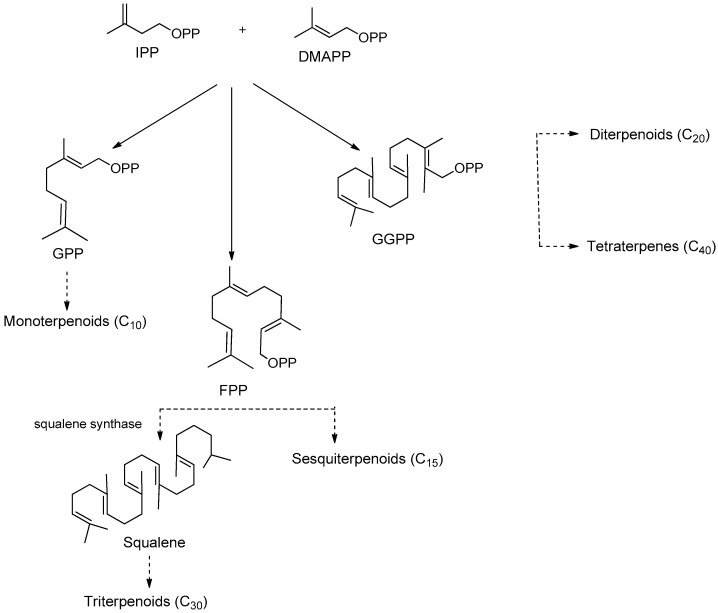
Simplified biosynthetic pathways of terpenes [123].

Terpenes, a group of secondary metabolites synthesized mainly by plants, represent one of the largest and most extensively studied classes of natural products, with over 40,000 known members. All terpenes are biosynthesized through condensation of the universal five-carbon building blocks isopentenyl diphosphate (IPP) with its allylic isomer dimethylallyl diphosphate (DMAPP) by the action of prenyltransferases to obtain the intermediated geranyl pyrophosphate (GPP, C_10_), farnesyl pyrophosphate (FPP, C_15_), or geranylgeranyl pyrophosphate (GGPP, C_20_). These intermediates can further undergo condensation and cyclization modifications to be converted into all terpenoid classes (Figure 13) [121,122]. Based on the number of isoprene units, terpenes can be classified as hemiterpenes (C_5_), monoterpenes (C_10_), sesquiterpenes (C_15_), diterpenes (C_20_), triterpenes (C_30_), and tetraterpenes (C_40_). The oxo-functionalization of these hydrocarbon compounds gave rise to terpenoids [123].

This class of NPs shows a wide array of pharmacological activities, including anticancer properties [79,124]. However, the clinical application of terpenes has been hampered by their moderate potency, limited water solubility, rapid metabolism, and low bioavailability. Chemical derivatization has been widely used as a strategy for improving not only the biological activity, but also the pharmacokinetic properties of these NPs. Using this approach, terpenes have been used as attractive starting points to obtain new compounds with potential to overcome the multidrug resistance in cancer [125,126,127].

### 3.1. Sesquiterpenoids

Dihydro-β-agarofuran sesquiterpenes were found to reverse the MDR phenotype of cells overexpressing P-gp [128]. In this regard, Callies et al. prepared a series (81 compounds) of semisynthetic derivatives starting from the natural dihydro-β-agarofuran sesquiterpene (**100**) and evaluate their ability to inhibit P-gp-mediated daunomycin efflux in MDR NIH-3T3MDR1 G-185 murine cells (Table 11). The influence of diverse structural features on P-gp inhibitory activity was explored, including ester moieties, anionic substituents, hydrogen bonding, π-interactions, hybridization and the presence of nitrogen atoms. Among all derivatives evaluated, compounds **101**–**105** displayed improved activity profile when compared to the lead compound (**100**) and verapamil (**1**) to reverse P-gp-mediated daunomycin and vinblastine resistance on MDR cells (Table 11). Interestingly, four of those derivatives (**102**–**105**) possess nitrogen-bearing substituents. Based on SAR studies, the authors suggested that the side chain length and the introduction of nitrogen on the ester moiety are key structural features for P-gp inhibition, giving rise to potent derivatives displaying submicromolar activities, as in the abovementioned compounds [129].

### 3.2. Diterpenoids

Helioscopinolide E (**106**), an ent-abietane diterpene isolated from *Euphorbia pedroi*, has shown an interesting P-gp efflux modulatory activity at 20 µM, in P-gp-transfected mouse T-lymphoma (L5178Y-MDR) cells. Aiming at potentiating its MDR reversal capabilities, Ferreira, et. al. derivatized compound **106** by introducing nitrogen-containing and aromatic moieties at C-3 and evaluated their P-gp modulatory properties, presenting the results as fluorescence activity ratio values (FAR, Table 12). The introduction of the oxime moiety in compound **107** resulted in a decrease of the P-gp modulatory activity (FAR reduced from 10.23 in helioscopinolide E, **106**, to 4.88). However, in derivatives **108**–**110**, the introduction of acetyl, benzoyl and 2-furoyl moieties, respectively, gave origin to compounds that displayed considerable modulation of the rhodamine 123 efflux by P-gp. Compound **109**, with a FAR of 56.37 was the most active compound and presented a strong synergistic effect with doxorubicin with a combination index (CI) of 0.152 (combination index values obtained using the Chou-Talalay method [130]. CI values <1 indicate the existence of synergism). Furthermore, molecular docking studies revealed that compound **109** bounds with high affinity to P-gp via π-π stacking or CH-π interactions and suggested that this compound may act as a noncompetitive modulator and impair the conformational changes requested for the efflux motion [82].

### 3.3. Triterpenoids

23-Hydroxybetulinic acid (**111**, Table 13), a natural lupane-type triterpene isolated from the traditional Chinese herb *Pulsatill chinensis,* has been reported for its anticancer effects and ability to re-sensitize MDR cancer cells to chemotherapy. This triterpene was used as starting point to prepare the derivative **112** (Table 13)**,** bearing a 1,4′-bipiperidine-1-carbonyl moiety at C-23 position. Compound **112** was found to markedly reduce resistance of HepG2/ADM, MCF-7/ADR, and KB-C2 MDR cells to P-gp substrates, while slightly reversed BCRP-mediated resistance to SN-38 and did not affect the MRP1-mediated MDR. These results suggested that **112** is a specific P-gp reversal agent. According to SAR studies, the introduction of a bulky hydrophilic moiety into C-23 contributed to the excellent MDR reversal activity. Further studies revealed that **112** did not affect mRNA or P-gp expression levels but modulated its ATPase activity. This derivative was also found to be able to modulate other MDR-associated proteins, such as ERK1/2 and AKT by inhibiting their phosphorylation. Moreover, 15 mg/kg of **112** significantly enhanced the anticancer effect of paclitaxel in a KB-C2 cell xenograft mice model, without affecting the body weight [131].

To further explore the potential of 1,4′-bipiperidine 23-hydroxybetulinic acid (**111**) derivatives, the same research group synthesized 17-bipiperidinyl derivatives **113** and **114** (Table 13) and evaluated their MDR-reversal properties. Both derivatives significantly increased the toxicities of vincristine and paclitaxel against HepG2/ADM and MCF-7/ADR P-gp overexpressing cancer cell lines, exhibiting stronger chemoreversal effects than **112** and verapamil (**1**) in both cell lines. These derivatives did not alter the mRNA or protein expression levels of P-gp, but significantly inhibited its drug efflux function and ATPase activity. Interestingly, docking analysis revealed that the bipiperidinyl group of **114** was superimposed with verapamil (**1**) at the P-gp active pocket, suggesting that the bipiperidinyl group with two basic nitrogen atoms may be important for the interaction of this derivative with the protein [132].

**Figure 14:**
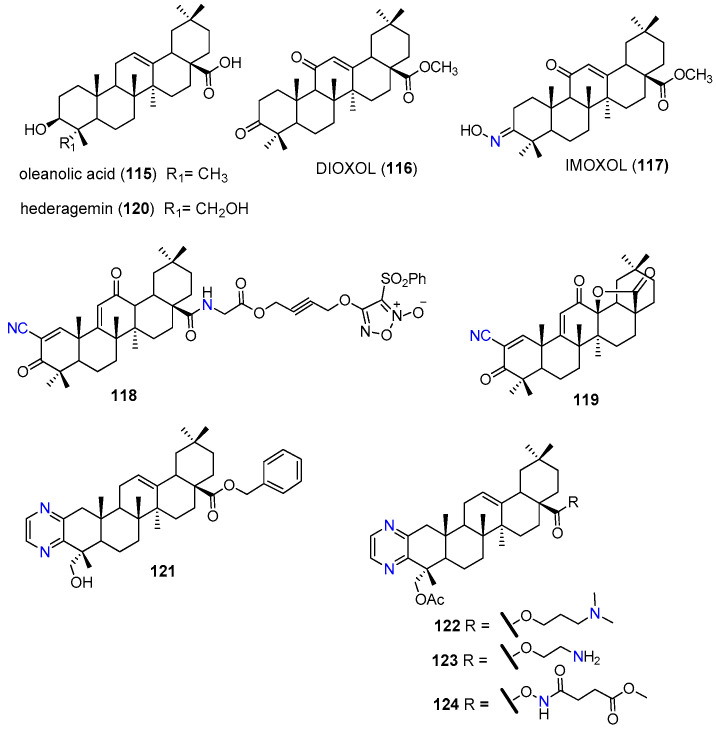
Triterpenoid nitrogen-containing derivatives **115**–**124.**

Oleanolic acid (**115**, Figure 14) is an oleanane-type triterpenoid that can be found in several plant species and in the human diet. This natural compound has been extensively reported for its cytotoxic and MDR reversal properties [133,134]. Oleanolic acid (**115**) has been frequently modified at C-3, C-11, C-12 and C-28 positions to prepare semisynthetic derivatives with improved pharmacological properties. Aiming at finding effective MDR modulators, Paszel-Jaworska et al. prepared and tested a series of oleanolic acid derivatives, from which DIOXOL (**116**) and IMOXOL (**117**) (Figure 14) exhibited interesting results. Both derivatives have shown to induce apoptosis of both sensitive (HL-60) and MRP1-overexpression multidrug resistant (HL-60/AR) leukemia cells. However, only IMOXOL (**117**) was effective in reducing the expression levels and transport function of MRP1 (Table 14). It must be emphasized that this derivative was more active than the known MRP1 inhibitor MK571. These results highlighted the influence of the replacement of hydroxyl group at C-3 with a hydroxyimino-moiety for the MRP1 inhibitory effects. According to the authors speculations, the spatial configuration of the oxime group at C-3 of IMOXOL (**117**) and the size and shape of the entire molecule allow the interaction of the compound with the ATP-binding sites of MRP1, resulting in the inhibition of the transporter [135].

Another oleanolic acid (**115**) derivative explored for its ability to reverse MDR was the amino acid-furoxan trihybrid **118 (**Figure 14), which inhibited cell proliferation and trigger apoptosis of both drug-sensitive (HCT-8, GI_50_ = 0.294 μM) and drug-resistant colon cancer cells (HCT-8/5-FU, GI_50_ = 0.232 μM). Treatment of HCT-8/5-FU cells with **118** significantly reduced the relative levels of P-gp, MRP1, and BCRP expression in a dose-dependent manner (Table 14). This effect was probably associated with high levels of nitric oxide production, induced by **118**, which is responsible for the nitration of critical tyrosine residues of P-gp, MRP1 and BCRP efflux pumps. In addition, a significant reduction of the tumor size was observed in BALB/c nude mice inoculated subcutaneously with HCT-8/5-FU cells and treated by gavage with **118** at 30 mg/kg [136].

Recently, the oleanolic acid derivative **119** was evaluated by Bin Zhu et. al. in multidrug resistant (A549/CDDP) and non-resistant (A549) lung cancer cells. This derivative exerted similar growth inhibitory effects on both cell lines with GI_50_ values of 2.39 µM and 1.96 µM, respectively. Compound **119,** at 2.5 µM, strongly inhibited the transport of rhodamine123 by P-gp at larger extent than did 5 µM of verapamil (**1**). Also, this compound markedly reduced the mRNA and protein expression levels of P-gp, likely due to the inhibition of NF-κB, MEK/ERK and PI3K/AKT signaling pathways (Table 14). Additionally, **119** revealed to be a multifunctional compound that targeted other MDR related proteins, namely by inhibiting thioredoxin reductase (TrxR) expression and activity. As a result of these multiple targeting properties, compound **119** not only suppressed cisplatin resistance in A549/CDDP cells but also could reverse the MDR caused by P-gp [137].

The natural pentacyclic triterpene hederagenin (**120**, (3β)-3,23-dihydroxy olean-12-en-28-oic acid, Figure 14), mainly extracted from *Hedera nepalensis var. sinensis* and *Hedera helix.*, is an oxidized derivative of oleanolic acid (**115**). This compound has been attracting a great deal of attention, due to its wide range of biological activities, including multidrug resistance reversal properties [140,141]. Main chemical modifications explored so far on hederagenin (**120**) scaffold are mostly focused at the C-3 and C-23 hydroxyl groups, the carboxylic acid group at C-28 and at the double bound between C-12 and C-13. Liu et. al. introduced side chains containing multiple nitrogen atoms at C-23 obtaining a series of derivatives with enhanced anticancer activity [142]. Among the derivatives prepared, the pyrazine derivative **121** (Figure 14) displayed a promising ability to reverse P-gp mediated MDR in vitro by sensitizing multidrug resistant oral epidermoid carcinoma (KBV) and human breast cancer (MCF7/T) cells to paclitaxel and vincristine, in a similar extent to verapamil (**1**) **(**Table 14**)**. This derivative was found to inhibit the P-gp efflux transport, as suggested by the rhodamine 123 assay, but increased the P-gp ATPase activity. The MDR reversal properties of **121** were confirmed in vivo, since it enhanced the anti-tumor activity of paclitaxel in a KBV xenograft tumor model in nude mice [138].

Driven by these good results, a series of **121** analogues (e.g **122**–**124**, Figure 14) were prepared aiming at improving not only the P-gp inhibitory properties, but also increasing the reduced solubility caused by the benzyl group at C-28. Derivatives **122**–**124** presented interesting P-gp modulating properties when tested in KBV cells. The common structural features of these compounds are the presence of nitrogen-bearing moieties at C-28 and the ring-A fused pyrazine, thus, the authors suggested that these could be key structural features for the chemoreversal effects. The most active derivative **122** increased the chemosensitivity of KBV cells towards paclitaxel at larger extent than **121** and the standard P-gp modulators verapamil (**1**), tariquidar (**10**) and zosuquidar (**11**). In vivo assay revealed that **122** also enhanced the efficacy of paclitaxel against KBV cancer cell-derived xenograft tumors (Table 14) [139].

**Figure 15:**
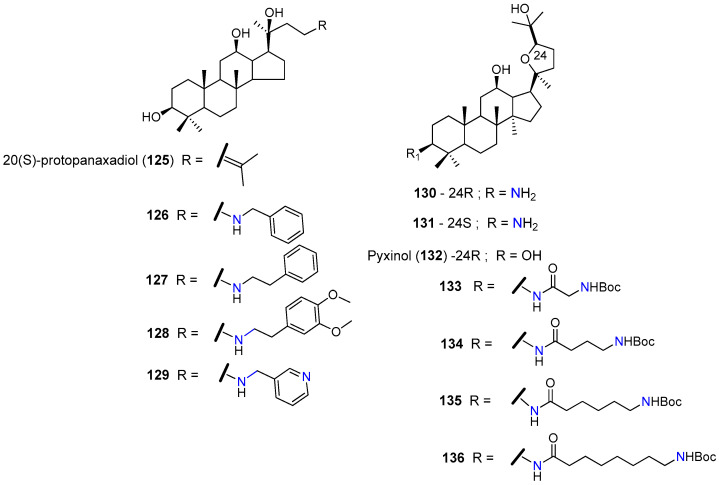
Triterpenoid nitrogen containing derivatives **125**–**136.**

Other triterpenoid that attracted the attention of medicinal chemists, due to its ability to significantly reverse P-gp-mediated MDR, was the 20(*S*)-protopanaxadiol (**125**, Figure 15), a dammarane-type triterpenoid derived from ginsenoside Rg3 [143]. Liu and co-workers synthesized a series of amine-substituted 20(*S*)-protopanaxadiol (**125**) analogues (e.g., **126**–**129**, Figure 15) based on the evidence that many P-gp inhibitors have common structural features such as lipophilic moieties and the presence of protonatable nitrogens. When tested in P-gp overexpressing KBvcr cells, compounds **126**–**129,** bearing aromatic–aliphatic amine substituents, displayed potent reversal activity against docetaxel, vincristine, and doxorubicin resistance. The best results were obtained with the unsubstituted aromatic amine derivatives **126** and **127**; both compounds revealed to be 1.3-2.6-fold more actives than verapamil (**1**) (Table 15).

These effects could be associated with the inhibition of P-gp, as suggested by the increased intracellular accumulation of the P-gp substrates doxorubicin and rhodamine 123 observed, after treatment with these derivatives [144]. No toxicity was observed for derivative **126** at 5 µM against both MDR and sensitive cells [144]. The oral administration of **126** at 100 mg/kg to nude mice bearing KB/VCR xenografts significantly enhanced the effect of doxorubicin, and partially reduced cardio and kidney toxicity associated with this drug [145,146]. Further in vivo studies revealed that this compound is well tolerated at high doses (1 g/kg) administered by gavage to a mouse model. Finally, **126** presented an excellent oral bioavailability of nearly 100%, and important propriety for further clinical development [146].

Ocotillol-type triterpenes have been reported to be potential P-gp modulators. Accordingly, Zhang et. al. explored the influence of amino moiety, by preparing the 3-amino 24(*R/S*)-epimer derivatives **130** and **131** and evaluating their chemosensitizing effects. While the treatment of P-gp-overexpressing SW620/Ad300 and HEK/ABCB1 cells with the 24(*R*)-epimer **130,** at 1 µM, significantly increased their sensitivity to paclitaxel and vincristine, treatment with its 24(*S*)-epimer **131** displayed only moderate activity (Table 15). Additionally, compound **130**, at 3 µM, was able to selectively inhibit P-gp activity (no effects observed against BCRP or MRP1) without affecting the protein expression levels. Molecular docking studies suggested that **130** bounded nicely to drug binding site of P-gp via both hydrogen and cation-π interactions, and reverse P-gp-mediated MDR by competitively inhibiting the pump drug efflux function [147].

Ren et. al. also prepared series of new amide ocotillol-type triterpenoid derivatives starting from the natural triterpenoid pyxinol (**132**) (e.g., **133**–**136**, Figure 15). The introduction of a linear alkyl amide, bearing a terminal Boc-protected amine at C-3, seemed to be a key factor for the MDR reversal activity. Compound **135** showed the strongest P-gp modulating properties by significantly reducing 28-fold the IC_50_ of paclitaxel in KBV cells (Table 15). The oral administration of **135** (15 mg/kg) significantly increased the antitumor activity of paclitaxel on a nude mouse bearing KBV xenografts, confirming in vivo its ability to reverse P-gp-related multidrug resistance [148].

## 4. Conclusions and Future Perspectives

Success in cancer chemotherapy has been hampered by the phenomenon of multidrug resistance. Diverse mechanisms have been identified in the MDR phenotype, including drug-efflux by ABC transporter proteins, which were identified to play a key role on MDR. Several strategies have been developed to overcome ABC transporters-mediated MDR, namely the inhibition of the efflux pump activity of the three main proteins involved, P-gp, MRP1 and BCRP. Although the promising results obtained *in vitro*, they have been disappointing in clinical terms with no inhibitor approved for clinical use so far. As a result, natural products, due to their exceptionally wide chemical diversity, coupled with their bioactivity, have been emerged as an encouraging alternative for the development of effective MDR reversers. Among natural product-derived compounds, special attention has been addressed to flavonoids and terpenoids as MDR reversers in cancer. Aiming at optimizing the structures of these secondary metabolites as MDR reversers, the structural modifications of their scaffolds has been addressed, namely by introduction of nitrogen-bearing moieties, which have been considered as important features in the interaction with ABC transporter proteins.

In relation to flavonoids, the introduction of nitrogen-bearing groups, such as aliphatic, aromatic and heterocyclic amines, amides and related functional groups into the chalcone backbone are among the most explored modifications reported so far. These modifications, in conjugation with the introduction of methoxy motifs on either A or B ring, seem to contribute for the establishment of strong interactions with ABC transporters. These strong interactions resulted in potent BCRP or P-gp modulation properties. Some derivatives displayed the ability to modulate the activity of both proteins and thus represent a good starting point for the development of dual P-gp/BCRP modulators. On the other hand, the synthesis of homo- and heterodimer derivatives, bearing linkers with nitrogen atoms, has been extensively explored on the flavone scaffold. The nature and the length of the linker apparently are the key factors for the selectivity of the modulators among ABC efflux pumps. Additionally, it should be highlighted that some of these derivatives exhibited improved water solubility, an important characteristic for the transition into a clinical drug candidate. However, considering the results from the flavonoid dimers, more studies are imperative for a better picture of the influence of the nitrogen-containing linker on the potency and selectivity of the derivatives over the ABC transporters, as well as on their pharmacokinetic properties. Regarding the flavonol and flavanol scaffolds, there is still much chemical space unexplored, and new chemical modifications should be performed to clarify the impact that the introduction of nitrogen-bearing groups has on their MDR reversal properties.

Concerning terpenoids, the triterpenes are by far the most explored regarding the introduction of nitrogen-containing moieties into the natural scaffolds. Novel lupane-, oleanane-, and dammarane- type triterpenoid derivatives, bearing nitrogen motifs, have displayed strong MDR reversal properties, mainly associated with P-gp modulation. In fact, the studies reviewed revealed that the effects of these triterpenoid derivatives over BCRP and MRP1 activities have been less explored when compared to P-gp, remaining poorly exploited.

As can be observed throughout this review, the introduction of nitrogen-bearing moieties into the flavonoid and terpenoid scaffolds have shown a positive impact in reversing the ABC transporters-mediated MDR. Many of the compounds reviewed are in good agreement with several studies that highlighted the presence of basic nitrogen atoms as relevant for the modulatory activity. However, several other derivatives described bear non-basic nitrogen atoms and still presented potent MDR reversal effects. Thus, the basicity of nitrogen atom is a relevant factor for the modulatory activity, mainly towards P-gp, due to its ability to act as a strong hydrogen bond-accepting moiety in the molecule, but it is not an essential structural requirement.

In this context, it is important to move forward with novel nitrogen-containing flavonoids and terpenoid libraries in order to provide more detailed and insightful SAR, regarding the modulation of ABC transporters function, and thus allowing a more rational design in the quest of MDR reversers, taking advantage of the recently published crystallographic structures of BCRP, P-gp, and MRP1, which strengthened the knowledge on the proteins function at molecular level.

Considering the multitarget nature of flavonoid and terpenoid scaffolds, it is also important to further explore the mechanisms of action of the new semisynthetic derivatives and identify the existence of targets other than ABC proteins that also contribute for the reversal of the complex MDR phenomenon. In addition, more bioavailability studies are warranted to bring these molecules into clinical use and hence increase the possibilities to overcome multidrug resistance in cancer.

## Figures and Tables

**Table 1:** Selected ABC protein inhibitors evaluated in clinical trials.

Inhibitor	Target Protein	Clinical Trial Phase	State	Ref.
Valspodar (PSC 833, **5**)	P-gp	III	Completed 1997–2000	[53]
Biricodar (VX-710, **7**)	P-gp/MRP1	II	Terminated 1998–2001	[54]
Tariquidar (XR9576, **10**)	P-gp	II	Completed 2003–2009	[55]
Zozuquidar (LY335979, **11**)	P-gp	III	Completed 2002–2009	[56]
Elacridar (GF120918, **12**)	BCRP	I	Completed 2002–2004	[57]
Dofequidar (MS-209, **13**)	P-gp/MRP1	III	Completed	[40]
Sulindac (**14**)	MRP1	II	Completed 2008–2012	[58]
Tetrandrine/CBT1^®^ (**15**)	P-gp	I	Ongoing. Start in March 2018	[59]
Erlotinib (**16**)	BCRP	I	Completed 2006–2009	[60]
Lapatinib (**17**)	BCRP	II	Completed 2007–2009	[61]

**Table 2:** BCRP inhibitory effects of compounds **38**–**44** in HEK293-ABCG2 cells.

Compound (Quinoxaline B Ring)	IC_50_ (µM) ^1^	Compound (2-naphthyl B Ring)	IC_50_ (µM) ^1^	Compound (3,4-methylene-dioxyphenyl B Ring)	IC_50_ (µM) ^1^
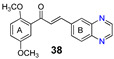	1.7	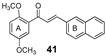	17.0	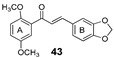	5.6
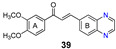	1.9	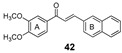	4.1	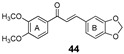	3.5
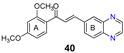	1.4	-		-	

^1^ IC_50_—Concentration producing 50% BCRP inhibition. The efficiency of each chalcone derivative to inhibit mitoxantrone efflux from BCRP-transfected HEK-293 cells was determined by flow cytometry, relatively to control HEK-293 cells transfected by the empty pcDNA 3.1 vector giving maximal mitoxantrone accumulation; The IC_50_ values were determined by using increasing inhibitor concentrations up to 20 μM or 50 μM. Lower IC_50_ values mean stronger BCRP inhibitory properties.

**Table 3:** Inhibitory activities of compounds **45**–**59** against BCRP and P-gp as determined in the pheophorbide A and calcein AM assays.

Compound	Cell Line and Assay/IC_50_^1^ (µM)			Cell Line/GI_50_^2^		Ref.
	MDCK II BCRP *Pheo. A*	MDCK II BCRP *Hoechst 33342*	P-gp Overexpressing A2780adr *Calcein AM*	MDCK II Wild Type	BDCK II BCRP	
**45**	1.30	-	0.42	3.42	5.14	[100]
**46**	0.60	-	0.48	12.60	10.90	[100]
**47**	0.84	-	2.34	-	-	[100]
**48**	0.29	-	18.8	3.46	4.80	[100]
**49**	0.19	-	14.9	132	92.9	[100]
**50**	2.18	-	-	-	-	[101]
**51**	1.30	-	-	7.88	7.08	[101]
**52**	0.98	0.50	-	17.40	20.50	[101]
**53**	0.97	0.57	0.49	69.30	65.90	[101]
**54**	0.60	0.50	-	89.40	93.20	[101]
**55**	-	0.22	1.13	80.00	78.00	[102]
**56**	-	0.88	-	-	-	[102]
**57**	1.97	-	-	7.56	9.38	[103]
**58**	6.33	-	-	-	-	[103]
**59**	3.37	-	-	18.90	27.7	[103]
Ko143 (**9**)	0.2	0.22	-	11.10	10.9	[100,101]
cyclosporine A (**2**)	-	-	1.41			[100]

^1^ IC_50_—Concentration producing 50% protein function inhibition. The efficiency of each chalcone derivative to inhibit substrate efflux from BCRP- or P-gp-overexpressing cells was determined by flow cytometry, relatively to control cells giving maximal substrate accumulation; The IC_50_ values were determined by using increasing concentrations of test compounds. ^2^ GI_50_—Concentration of compound that inhibited cell growth by 50%, used to access intrinsic cytotoxicity of tested compounds determined by the MTT assay, using MDCK II BCRP and MDCK II wild-type cells.

**Table 4:**
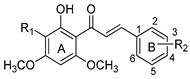
Doxorubicin-resistance reversal effects of chalcone derivatives **60**–**64** at a concentration of 12.5 µM, on P-gp-overexpressing MCF-7/DOX cells [84].

Compound	R1	R2	GI_50_ DOX (µM) ^1^	RF ^2^
**60**	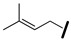	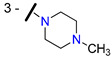	23.59	2.17
**61**	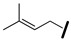	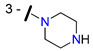	1.02	50.19
**62**	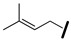	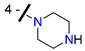	14.84	3.45
**63**	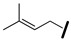	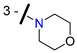	31.60	1.62
**64**	OH	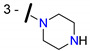	2.63	19.46

^1^ GI_50_—Defined as the concentration of doxorubicin required to obtain 50% cell growth inhibition, when cells were co-treated with the indicated derivatives at 12.5 µM. GI_50_ of doxorubicin alone = 51.59 µM; ^2^ RF—Reversal fold. RF is calculated as a ratio of GI_50_ of DOX alone and IC_50_ of DOX in the presence of tested compound at 12.5 µM. Higher RF values mean stronger MDR reversal effects.

**Table 5:**
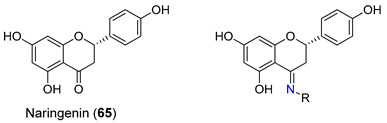
Inhibitory activity of naringenin derivatives **66**–**69** in HEK293-MDR (BCRP) and BHK21 (MRP1) cells [104].

Compound	R	Inhibition (%)	
		BCRP	MRP1
**Naringenin (65)**	-	26.6	8.91
**66**	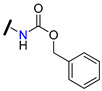	−2.77	62.6
**67**	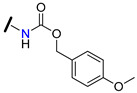	−0.05	95.0
**68**	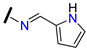	53.5	56.3
**69**	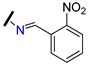	71.1	45.1
Ko143 (**9**) (1 µM)	-	100	-
Verapamil (**1**) (35 µM)	-	-	100

**Table 6:** Effects of flavone dimers **75**–**79** in modulating MDR in multidrug resistant cancer cells in vitro.

Compound	Cell Line/ABC Protein-Mediated Resistance EC_50_ (µM) ^1^				Cell Line /GI_50_ (µM) ^2^	Ref.
	LCC6MDR P-gp/Paclitaxel Resistance	MCF7-MX100 BCRP/Topotecan Resistance	HEK293/R2 BCRP/Topotecan Resistance	2008/MRP1 Doxorubicin Resistance	L929	
**75**	0.950	-	-	-	>100	[108,109]
**76**	0.222	-	-	-	>100	[108,110]
**77**	0.148	-	-	-	85.000	[108,111]
**78**	-	-	-	0.053	>100	[112]
**79**	>1000	0.001	0.002	>1000	>100	[113]
Verapamil **(1)**	0.428	-	-	-	89.200	[111]
Kol143 **(9)**	1.060	0.009	0.009	1.95	31.400	[113]

^1^—EC_50_-Defined as the concentration of flavonoid dimers needed to reduce the GI_50_ of anticancer agent against LCC6MDR cells by 50%. Lower EC_50_ values mean stronger MDR reversal properties; ^2^—GI_50_-Defined as the concentration of compound that inhibits cell growth by 50%.

**Table 7:**
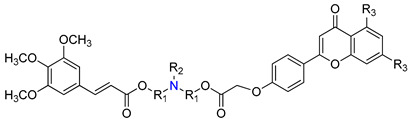
P-gp modulating activity of flavone derivatives **80**–**84** on K562/DOX cells [114].

Compound	R1	R2	R3	[I]_0.5_ (µM) ^1^	α_max_^2^
**80**	(CH_2_)_5_	CH_3_	OH	0.96	0.99
**81**	(CH_2_)_5_	CH_3_	H	0.34	0.99
**82**	(CH_2_)_5_	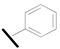	H	0.43	0.99
**83**	(CH_2_)_5_	H	H	1.32	0.69
**84**	(CH_2_)_2_O(CH_2_)_2_	CH_3_	H	1.23	0.86
Verapamil (**1**)	-	-	-	1.60	0.70

^1^ [I]_0.5_ represents the concentration that causes a half-maximal increase (a = 0.5) in the nuclear concentration of pirarubicin and measures the potency of the modulator. Lower [I]_0.5_ values mean stronger P-gp modulators; ^2^ α_max_ represents the efficacy of the modulator and is the maximum increase in the nuclear concentration of pirarubicin in resistant cells that can be obtained with a given compound. The value of α_max_ varies between 0 (in the absence of the inhibitor) and 1 (when the amount of pirarubicin in resistant cells is the same as in sensitive cells).

**Table 8:**
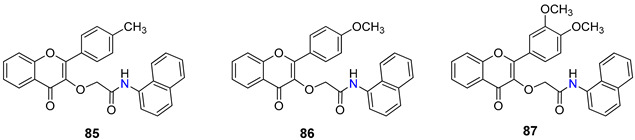
MDR reversal effects of flavone derivatives **85**–**87** against BCRP and P-gp [103].

Compound	IC_50_ (µM) ^1^		EC_50_ (µM) ^2^		GI_50_ (µM) ^3^				Therapeutic Ratio ^4^	
	MDCK II BCRP	A2780 adr (P-gp)	MDCK II BCRP	A2780 adr (P-gp)	MDCK II wt	BDCK II BCRP	A2780	A2780/ADR	BCRP	P-gp
**85**	-	1.89	-	5.01	-	-	68.7	98.6	-	52.2
**86**	5.09	1.41	0.793	-	>100	>100	-	-	>19.6	-
**87**	3.75	5.43	0.446	3.12	>100	>100	43.6	45.3	>26.7	8.3

^1^ IC_50_—Defined as the concentration of flavone derivative needed to produce 50% ABC protein function inhibition. Lower IC_50_ values mean stronger ABC protein inhibitors; ^2^ EC_50_—half-maximal MDR reversal concentrations. Defined as the concentration of flavone derivative needed to reduce the GI_50_ of the corresponding antineoplastic agent (daunorubicin (P-gp) or SN-38 (BCRP) by 50%. Lower EC_50_ values mean stronger MDR reversal properties values; ^3^ GI_50_—Defined as the concentration of derivative that inhibited cell growth by 50%. Lower GI_50_ values mean higher toxicity; ^4^ Therapeutic ratio—Ratio between toxic dose and therapeutic dose for that compound. Therapeutic ratio was calculated taking the IC_50_ as well as the GI_50_ values of the resistant cells into account (therapeutic ratio = IC_50_ in resistant cell line/GI_50_ in the same cell line). High therapeutic ratios are desirable.

**Table 9:**
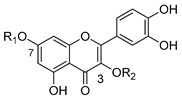
P-gp modulation activities of quercetin derivatives **88**–**91** in MES-SA/Dx5 cell line [118].

Compound	R_1_	R_2_	IC_50_ (µM) ^1^
**88**	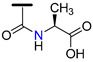	H	0.41
**89**	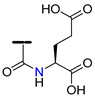	H	0.14
**90**	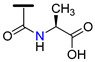	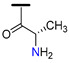	0.78
**91**	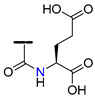	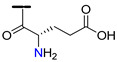	0.71

^1^ IC_50_—Defined as the concentration of derivative needed to produce 50% P-gp protein function inhibition. Lower IC_50_ values mean stronger P-gp protein inhibitors.

**Table 10:**
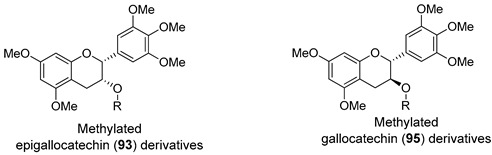
P-gp, BCRP and MRP1 reversal activity of epigallocatechin (**93**) and gallocatechin (**95**) derivatives **96**–**99** in LCC6MDR (P-gp), HEK293/R2 (BCRP) and 2008/MRP1 (MRP1) cells. Verapamil (**1**) (P-gp) and K0143 (**9**) were used as positive controls [107].

Compound	R	RF ^1^		
		LCC6MDR	HEK293/R2	2008/MRP1
**epigallocatechin-3-gallate (92)**	-	1.2	-	-
**Epigallocatechin Derivatives**				
**96**	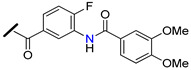	48.2	2.7	0.8
**97**	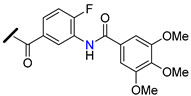	43.8	2.3	0.9
**Gallocatechin Derivatives**				
**98**	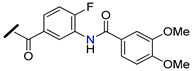	31.4	10.4	2.6
**99**	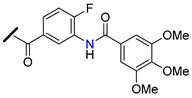	53.6	12.3	2.6
Verapamil (**1**)	3.8		-	-
K0143 (**9**)	-		19.5	-

^1^ Reversal fold (RF)—ratio between GI_50_ of paclitaxel in MDR cells in the absence and presence of 1 µM of the tested compounds. Higher RF values mean stronger ABC modulation properties.

**Table 11:**
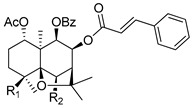
Reversal effects of sesquiterpene **100** and its derivatives **101**–**105** of P-gp-mediated daunomycin resistance on NIH-3T3MDR1 G-185 murine cells [129].

Compound	R1	R2	Activity		
			K_i_^1^ (µM)	RF ^2^ (Daunomycin)	RF ^2^ (Vimblastine)
**100**	OH	OH	0.28	5.6	23.7
**101**	OH	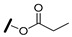	0.54	10.8	19.1
**102**	OH	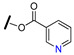	0.13	11.8	17.4
**103**	OH	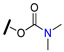	1.21	11.8	11.2
**104**	OH	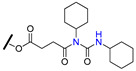	0.19	15.8	27.4
**105**	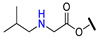	OH	0.63	10.0	20.6
Verapamil (**1**)	-	-		2.8	8.7

^1^ Ki—concentration of compounds that inhibits 50% of P-gp-mediated daunomycin transport; ^2^ RF—Reversal fold: indicate the ability of the tested compounds to reduce resistance of multidrug resistance cells to daunomycin/vinblastine. RF values were calculated by the ratio between IC_50_ of daunomycin or vinblastine in the absence and in the presence of the tested sesquiterpene derivatives at 1 µM.

**Table 12:**
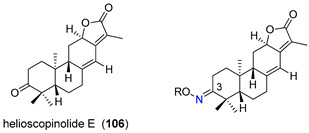
P-gp modulatory activity of helioscopolide E (**106**) and its derivatives **107**–**110** on mouse T-lymphoma P-gp-transfected L5178Y cells [82].

Compound at 20 µM	R	Fluorescence Activity Ratio (FAR) ^1^
Helioscopinolide E (**106**)	-	10.23
**107**	H	4.88
**108**	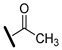	38.64
**109**	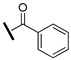	56.37
**110**	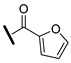	40.69
verapamil (**1**)	-	9.66

^1^ FAR—Fluorescence activity ratio: cytoplasmic accumulation ratio of Rhodamine 123 between L5178Y-MDR (resistant) and L5178Y-PAR (sensitive) cells. FAR > 1 reveals the existence of P-gp modulation, FAR > 10 means strong P-gp modulation.

**Table 13:**
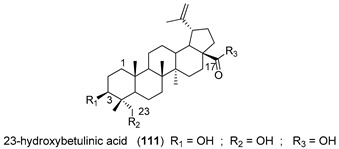
P-gp-resistance reversal properties of triterpene derivatives **112**–**114** [131,132].

Compound	R1	R2	R3	Cells/In Vivo Models	Biological Activity
**112**	OH	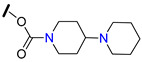	OH	HepG2/ADM	At 5 µM reduced the GI_50_^1^ of vincristine 132-fold and paclitaxel 79-fold. Suppressed P-gp efflux function. Inhibition of ERK1/2 and AKT phosphorylation.
				MCF-7/ADR	At 5 µM reduced the GI_50_^1^ of vincristine 151.04-fold and paclitaxel 151.07-fold.
				KB-C2 cell xenografts in nude mice model	At 15 mg/kg significantly enhanced the anticancer activity of paclitaxel (18 mg/kg), with no significant change in the body weight (Reduced toxicity).
**113**	OH	OH	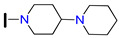	HepG2/ADM	At 4 µM reduced the GI_50_^1^ of vincristine 48.03–fold and paclitaxel 82-fold. Inhibited P-gp ATP-ase activity and suppress its efflux function
				MCF-7/ADR	At 4 µM reduced the GI_50_^1^ of vincristine 29-fold and paclitaxel 47-fold.
**114**	OAc	OAc	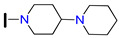	HepG2/ADM	At 4 µM reduced the GI_50_^1^ of vincristine 232-fold and paclitaxel 282-fold. Inhibited P-gp ATP-ase activity and suppress its efflux function.
				MCF-7/ADR	At 4 µM reduced the GI_50_^1^ of vincristine 183-fold and paclitaxel 59-fold.
verapamil (**1**)**^2^**	-	-	-	HepG2/ADM	At 5 µM reduced the GI_50_^1^ of vincristine 22-fold and paclitaxel 11-fold.
				MCF-7/ADR	At 5 µM reduced the GI_50_^1^ of vincristine 29-fold and paclitaxel 59-fold.

^1^ GI_50_—Defined as the concentration of compound that inhibited cell growth by 50%. Lower GI_50_ values mean higher intrinsic toxicity; ^2^ verapamil (**1**) was used as positive control.

**Table 14:** MDR reversal properties of oleanane-type triterpene derivatives **117**–**119, 121** and **122.**

Compound	ABC Protein Target	Cell Line/Animal Model	Activity/Mechanism of Action	Ref.
**117**	MRP1	HL-60/AR	Inhibited MRP1 transport function (short term response). Reduced the mNMR and protein expression levels of MRP1.	[135]
**118**	P-gp, MRP1 BCRP	HCT-8/5-FU	Reduced the relative levels of P-gp, MRP1, and BCRP by nitrating these cellular drug efflux proteins.	[136]
**119**	P-gp	A549/CDDP	Reduced the P-gp expression at the protein and mRNA levels. Suppressed P-gp ATPase activity. Inhibited MEK/ERK and PI3K/AKT pathways. Inhibited TrxR expression and activity.	[137]
**121**	P-gp	KBV	At 10 µM reduced the GI_50_^1^ of paclitaxel 158-fold and the GI_50_^1^ of vincristine 78-fold. Inhibited the P-gp efflux activity without affecting its protein expression. Increased P-gp ATP-ase activity.	[138]
		MCF7/T	At 10 µM reduced the GI_50_^1^ of paclitaxel 14-fold and the IC_50_ of vincristine 33-fold.	
		KBV xenograft in nude mice	At 10 mg/kg significantly enhanced anti-tumor activity of paclitaxel with good safety profile.	
**122**	P-gp	KBV	At 10 µM reduced the GI_50_^1^ of paclitaxel 80.6-fold Increased P-gp ATPase activity.	[139]
		KBV xenograft in nude mice	Enhanced antitumor activity of paclitaxel.	

^1^ GI_50_—Defined as the concentration of compound that inhibited cell growth by 50%. Lower GI_50_ values mean higher intrinsic toxicity.

**Table 15:** P-gp-resistance reversal properties of triterpene derivatives **126**, **127**, **130**, **131**, **135**.

Compound	Cells line/Animal Model	Activity/Mechanism of Action	Ref.
**126**	KBvcr cells	Sensitized multidrug-resistant cells to docetaxel, vincristine and doxorubicin. At 5 µM reduced the GI_50_^1^ of docetaxel 111-fold, the GI_50_ of vincristine 87-fold, and the GI_50_ of doxorubicin 56-fold. EC_50_^2^ = 1.367 µM for doxorubicin resistance. Inhibited P-gp–efflux function.	[144]
	KBvcr xenografts mice model	At 100 mg/kg significantly enhanced the inhibitory effect of doxorubicin against a multidrug-resistant tumor in the xenograft model.	[145,146]
**127**	KBvcr cells	Sensitized multidrug-resistant cells to docetaxel, vincristine and doxorubicin. At 5 µM reduced the GI_50_^1^ of doxorubicin 196-fold, the GI_50_^1^ of vincristine 117-fold, and the GI_50_^a^ of Adriamycin 62-fold. EC_50_^2^ = 1.31 µM for doxorubicin resistance.	[144]
**130**	SW620/Ad300	At 3 µM significantly reversed the resistance to paclitaxel and vincristine by reducing the GI_50_^1^ of paclitaxel 26-fold and the GI_50_^1^ of vincristine 28-fold. Suppressed the efflux function of P-gp. Stimulated the ATPase activity of P-gp.	[147]
	HEK/ABCB1	At 3 µM reduced the GI_50_^a^ of paclitaxel 13.00-fold and the GI_50_^1^ of vincristine 5.58-fold.	
**131**	SW620/Ad300	At 3 µM reduced the GI_50_^1^ of paclitaxel 1.41-fold and the GI_50_^1^ of vincristine 1.81-fold.	[147]
**135**	KBV	At 5 µM reduced the GI_50_^1^ of paclitaxel 28-fold. Significantly increased the population of paclitaxel-treated cells in the G2-M phase. Increased the accumulation of Rhodamine123 in KBV cells in a dose-dependent manner. Increased the P-gp-ATPase activity over the basal level by 6.03-fold.	[148]
	KBV xenograft nude mice model	Oral administration of 10 mg/kg, significantly increased the tumor inhibitory activity of paclitaxel (30 mg/kg).	

^1^ GI_50_—Defined as the concentration of compound that inhibited cell growth by 50%. Lower GI_50_ values mean higher intrinsic toxicity; ^2^ EC_50_—Defined as the concentration of derivative needed to reduce the GI_50_ of the corresponding antineoplastic agent by 50%. Lower EC_50_ values mean stronger MDR reversal properties values.
